# Angioprevention of Urologic Cancers by Plant-Derived Foods

**DOI:** 10.3390/pharmaceutics14020256

**Published:** 2022-01-21

**Authors:** Melissa García-Caballero, José Antonio Torres-Vargas, Ana Dácil Marrero, Beatriz Martínez-Poveda, Miguel Ángel Medina, Ana R. Quesada

**Affiliations:** 1 Department of Molecular Biology and Biochemistry, Faculty of Sciences, University of Malaga, Andalucía Tech, E-29071 Malaga, Spain; melissa@uma.es (M.G.-C.); torresvargas@uma.es (J.A.T.-V.); anadacil95@uma.es (A.D.M.); bmpoveda@uma.es (B.M.-P.); medina@uma.es (M.Á.M.); 2 IBIMA (Biomedical Research Institute of Malaga), E-29071 Malaga, Spain; 3 CIBER de Enfermedades Cardiovasculares (CIBERCV), E-28019 Madrid, Spain; 4 CIBER de Enfermedades Raras (CIBERER), E-29071 Malaga, Spain

**Keywords:** angiogenesis, chemoprevention, angioprevention, phytochemicals, urologic cancer, prostate cancer, bladder cancer, kidney cancer

## Abstract

The number of cancer cases worldwide keeps growing unstoppably, despite the undeniable advances achieved by basic research and clinical practice. Urologic tumors, including some as prevalent as prostate, bladder or kidney tumors, are no exceptions to this rule. Moreover, the fact that many of these tumors are detected in early stages lengthens the duration of their treatment, with a significant increase in health care costs. In this scenario, prevention offers the most cost-effective long-term strategy for the global control of these diseases. Although specialized diets are not the only way to decrease the chances to develop cancer, epidemiological evidence support the role of certain plant-derived foods in the prevention of urologic cancer. In many cases, these plants are rich in antiangiogenic phytochemicals, which could be responsible for their protective or angiopreventive properties. Angiogenesis inhibition may contribute to slow down the progression of the tumor at very different stages and, for this reason, angiopreventive strategies could be implemented at different levels of chemoprevention, depending on the targeted population. In this review, epidemiological evidence supporting the role of certain plant-derived foods in urologic cancer prevention are presented, with particular emphasis on their content in bioactive phytochemicals that could be used in the angioprevention of cancer.

## 1. Introduction

The burden of cancer incidence and mortality is rapidly growing worldwide due to aging and growth of the population, and to changes in the prevalence of several risk factors, many of which might be associated with social and economic development. In most developed countries, cancer has now become the leading cause of premature death, surpassing cardiovascular diseases. Efforts to build a sustainable infrastructure for the dissemination of cancer prevention measures and provision of cancer is critical for the global control of this disease [1].

Urologic cancers affect the organs and structures of the male and female urinary tract (kidneys and bladder) and the male reproductive system (prostate, penile and testis). Although they may often share symptoms such as changes in urination and sexual function, they differ greatly in their prevalence, ranging from that of prostate cancer, the most frequently diagnosed cancer in men in over one-half of the countries of the world, to the very rare penile cancer [1].

With almost 1.4 million new cases and 375,000 deaths worldwide in 2020, prostate cancer is the second most frequent cancer and the fifth leading cause of cancer death among men [1]. Although relatively little is known about prostate cancer etiology, several risk factors have been identified, such as aging, family history, genetic mutations (e.g., BRCA1, BRCA2, ATM or HOXB13) and conditions (Lynch syndrome) [2]. In addition, there is growing evidence that some lifestyle and environmental factors, including smoking, excess body weight and some nutritional factors, can contribute to increase the risk of advanced prostate cancer. As most patients are normally asymptomatic at early stages, diagnosis is primarily based on the controversial use of prostate-specific antigen (PSA) testing for screening. When limited to the gland, prostate cancer is normally curable. On the contrary, the average five-year survival prognosis reaches only 30% of the cases in patients who have been diagnosed at advanced stages, frequently presenting metastatic dissemination of tumor cells [3].

Bladder cancer, affecting the cells that line the urinary bladder, is the 10th most frequently diagnosed cancer worldwide, with approximately 573,000 new cases and 213,000 deaths in 2020. It is more common in men, for whom it is the sixth most diagnosed cancer and the ninth leading cause of cancer death [1]. In developed countries, bladder cancer is the costliest highly prevalent cancer, due to the decrease in mortality derived from the improvements in treatment. Fortunately, the majority of patients are diagnosed at a highly treatable stage, what gives them a high life expectancy, but also requires a lengthy follow-up period of surveillance, the treatment of recurrences and the management of side effects and complications [4].

Kidney cancer is the 16th most commonly diagnosed cancer worldwide, with approximately 431,000 new cases and almost 180,000 deaths in 2020 [1]. It is the sixth most common cancer for men and the ninth most common cancer for women in the United States, with more than 76,000 new cases diagnosed each year in this country at an average age of 64. Those tumors with genetic predispositions (such as von Hippel–Lindau disease) are diagnosed 20 years earlier. The most common type of kidney cancer is renal cell carcinoma (RCC), which accounts for almost 90% of kidney cancer cases, and develops in the proximal renal tubules, a region of the nephron responsible for many of the homeostatic properties of the kidney. Among more than ten subtypes of RCC tumors, the clear cell RCC is the most common and responsible for the majority of deaths from kidney cancer [5]. Survival rates depend on several factors, including the type of tumor, the affected cell types and the stage of the cancer when it is first diagnosed. The 5-year survival rate ranges from 93% for those who are diagnosed when the cancer is located only in the kidney, to as low as 13% for those others in which the cancer has spread to a distant part of the body. Modifiable risk factors for RCC include smoking, obesity, hypertension, diet and alcohol use. Prevention strategies include improving the access to regular healthcare, facilitating earlier diagnosis, and addressing lifestyle factors [6].

Testicular cancer is quite unusual, compared with the above-mentioned urologic cancers, since accounts for just 1% of all cancers that occur in men. Nevertheless, it is more prevalent in younger men, and has become the most common type of cancer affecting men between 15 and 34 years old [7]. Alarmingly, the number of cases with this type of cancer diagnosed yearly is on the rise in Western countries. Although recent studies have broadened our knowledge on the risk factors for testicular cancer, the underlying reasons for this increasing incidence remain elusive [8]. Finally, the least common urologic tumor is penile cancer, which is considered a rare malignancy due to its low incidence. Penile squamous cell carcinoma accounts for over 95% of penile malignancies, with about half of the cases linked to human papilloma virus infection [9,10]. With less than one case per 100,000 men in developed countries, the incidence of penile cancer is significantly higher in developing countries. Diagnosis of penile cancer is often delayed due to lack of awareness and significant social and psychological stigma, what contributes to a reduced survival, barely reaching 50% for 5-year survival in an overall stage-independent analysis [11].

Fortunately, high fractions of the most prevalent urologic cancers are detected at their early stages, what is essential to improving outcomes. Current guidelines suggest that in a significant proportion a radical treatment is not required, and recommend informed/shared decision-making, after which a patient could choose the option of active surveillance. Active surveillance relies on a closely monitoring of the disease evolution, avoiding or delaying the use of treatments such as radiation therapy or surgery, and their life-altering side effects, until they are absolutely necessary because the cancer progresses. In the context of prostate cancer, active surveillance has become increasingly popular as a management option for localized low risk tumors [12]. With an estimated prevalence that can reach up to 60% in men at the of age of 80 [13], and given that most of the tumors are slow-growing, doctors frequently opt to preserve the quality of life of the prostate cancer patients and recommend active surveillance [14]. Active surveillance is also gaining recognition as a safe initial management approach for well-selected patients with clinically localized kidney cancer [15,16] and low-grade bladder tumors [17].

In many cases, patients who are being monitored through active surveillance seek a more proactive approach, being receptive to assume changes in their lifestyle or use preventive drugs that could lead to slow the disease progression. This is the main idea behind cancer chemoprevention, a term firstly defined by Michael B. Sporn in 1976, aimed to use agents that do not kill healthy cells to prevent, arrest or reverse the progression of premalignant cells towards full malignancy [18].

There are different levels of cancer prevention, depending on their targeted population. Primary prevention aims to decrease the global incidence of disease in healthy population. Secondary prevention aims to reduce the impact of a particular cancer in higher-risk populations, with actions focused to detect and treat the disease as soon as possible to halt or slow its progress, even before symptoms appear. Tertiary prevention aims to soften the impact of more advanced stages of the disease, to improve the results of the oncologic treatment, their survival rate and the patients’ quality of life. In spite of some controversial results obtained in some reported preventive interventions, it is becoming increasingly clear that successful interventions for modifiable lifestyle factors should minimize economic burdens and deliver efficient care to relatively large patient populations [19].

## 2. Physiological and Pathological Angiogenesis

### 2.1. The Angiogenic Process

Angiogenesis refers to the generation of new blood vessels from a pre-existing vascular bed [20]. A well-regulated angiogenic process is a hallmark of several physiological conditions such as embryonic development, tissue repair and ovulation [21]. However, abnormal angiogenesis is also the basis of numerous pathological processes including atherosclerosis, chronic inflammation, tumor growth and metastasis [22,23,24,25,26]. These physiological and pathological conditions have some common angiogenic characteristics, but depending on the specific organ or disease under investigation, the angiogenic process might display differences due to the organ-specific endothelial cell heterogeneity associated with different vascular beds and specialized functions of these tissues [27].

Angiogenesis is a dynamic process involving important changes to favor the transition of endothelial cells from a quiescent to an activated state [28]. First, angiogenic stimuli such as hypoxia or proangiogenic chemokines lead to the blood vessel dilation and increased permeability. Consequently, the detachment of pericytes and degradation of basement membrane and extracellular matrix (ECM) takes place. Then, other angiogenic factors promote endothelial cell proliferation and migration to initiate a new sprout. Once this is accomplished, endothelial cells present in the newly formed vessel experience phenotypic adaptations to form tip and stalk cells, where tip cells are the non-proliferating cells specialized in sensing their environment and guiding the new sprout, and stalk cells are the less migratory and more proliferative cells elongating the nascent vessel [29,30]. New vessels are connected through anastomosis and develop lumens that are surrounded by a basement membrane. In parallel, mesenchymal cells differentiate into smooth muscle cells and pericytes that participate in the subsequent stabilization of the new blood vessel. During vessel stabilization, the junctional integrity, mechanical strength and tightness of the endothelium is re-established, permeability is controlled and blood flow starts in the mature stable vessel [31].

### 2.2. Molecular Mechanisms of Angiogenesis

At the molecular level, the formation of new blood vessels is regulated by many pro- and antiangiogenic factors, which in turn, control the angiogenic switch and tip the balance by either promoting or inhibiting the angiogenic process [32]. Many different proteins have been identified as angiogenesis activators, including vascular endothelial growth factor (VEGF), basic fibroblast growth factor (bFGF), angiopoietins, platelet-derived endothelial growth factor (PDGF), tumor necrosis factor (TNF)-α, granulocyte colony-stimulating factor (G-CSF), platelet-derived endothelial growth factor (PDGF), placental growth factor (PlGF), transforming growth factor (TGF), interleukin-8 (IL-8), hepatocyte growth factor (HGF), epidermal growth factor (EGF), C-C motif chemokine ligand 2 (CCL2), such as domain multiple 6 (EGFL6), endothelins, hypoxia-inducible factors (HIF1), insulin-like growth factor 1 (IGF1) and matrix metalloproteinases (MMPs), among others. In physiology, angiogenesis activators are balanced by endogenous angiogenesis inhibitors, including angiostatin, endostatin, thrombospondin-1, interferons, IL-1, IL-12, metalloproteinase inhibitors and retinoic acid, among others (reviewed in [32,33,34,35]).

**Figure 1:**
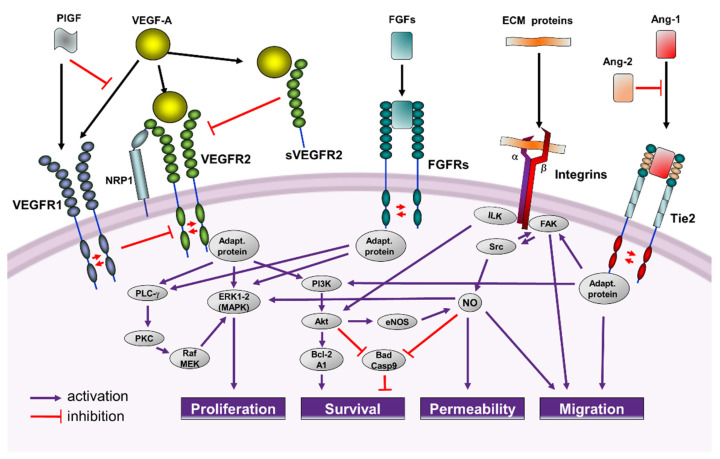
Molecular mechanisms of angiogenesis in activated endothelial cells. Ligand binding induces dimerization and autophosphorylation of tyrosine kinase receptors (VEGFR2, FGFRs, Tie-2). Receptor activation brings on the recruitment of several adaptor proteins that trigger signaling pathways leading to proliferation, migration, improved survival and loss of intercellular adhesions of endothelial cells.

The VEGF family deserves a special mention. It comprises different ligands (VEGF-A to -E and PlGF) that bind to their receptor (VEGFR), leading to receptor dimerization and autophosphorylation of the intracytoplasmic domains in specific tyrosine residues. The discovery of VEGF-A as a potent endothelial cell-specific mitogen that stimulates endothelial cell proliferation, survival and microvascular permeability, was a milestone in the vascular field [36]. Binding of VEGF-A to the receptor VEGFR2 is the main extracellular signal triggering an angiogenic response in endothelial cells, and it is considered to be responsible for the connection of the angiogenic switch in cancer and other angiogenesis-dependent diseases [37]. VEGFR1 acts as a trap for VEGF-A, preventing its binding to VEGFR2. PlGF, which only binds VEGFR1, can inhibit this trap, increasing the bioavailability of VEGF-A for VEGFR2 activation [34]. VEGF-A/VEGFR2 mediated signaling, is enhanced by neuropilins (NRP), acting as co-receptors [38] (Figure 1).

The VEGFR2-induced proliferative pathway is mediated by activation of the ERK-MAPK cascade, which can also be amplified by nitric oxide (NO), whereas the PI3K/Akt signal transduction pathway is crucial in the processes leading to endothelial cell survival induced by VEGF-A [33].

In response to a VEGF-A gradient, upregulated Dll4 expression is detected in tip cells, and consequently, NOTCH pathway is activated in stalk cells, making them less responsive to the VEGF-A stimulus, by downregulation of VEGFR2 [39]. In addition to their role in angiogenesis, some members of the VEGF family and their receptors, such as VEGF-C and VEGFR3, are also involved in lymphangiogenesis [40], the formation of new lymphatic vessels.

The FGF superfamily, composed by about 20 proteins in mammals, directly stimulates FGFRs on endothelial cells or activates angiogenesis by promoting the production of angiogenic factors from other cell types [41]. Among the FGF family members, bFGF stands out for its relevant angiogenic activity. As for VEGFR, binding of bFGF to FGFR induces the receptor dimerization and cross-phosphorylation. This process is essential for the docking and activation of a number of effectors that will activate the ERK-MAPK pathway, leading to increased endothelial cells proliferation [33] (Figure 1). Interestingly, the angiogenic activity of bFGF can be affected by VEGF signaling, so that VEGF-A and bFGF act synergistically to promote tumor angiogenesis [34].

Angiopoietins (Ang-1 and -2) and their receptors, Tie 1 and 2, are important molecular players in the regulation of the angiogenic remodeling and vessel stabilization that take place after the VEGF-A action [42,43]. Once Tie2 receptor binds Ang-1, it becomes activated upon autophosphorylation at tyrosine residues, initiating the promigratory, prosurvival PI3K/Akt pathway in endothelial cells and promoting the vessel assembly and maturation by their association with mural cells [33] (Figure 1). The Tie2 signaling pathway is regulated with a high degree of spatial and temporal precision by an agonist-antagonist interplay. The localized expression of Ang-2, which is a weaker agonist of Tie2, inhibits the Ang-1-mediated Tie2 activation, priming the vascular endothelium to exogenous cytokines, such as VEGF-A. This differential regulation of angiopoietin binding allows for stimuli in the cellular microenvironment to potentially modify the Tie2-mediated signaling [43].

The interaction between mural and endothelial cells is important for the maturation, remodeling and maintenance of the vascular system via the secretion of growth factors or modulation of the ECM. Chemotactic and mitogenic activities mediated by the PDGF/PDGFR paracrine signaling loop are crucial for the formation, branching and maintenance of blood vessels. PDGFs, produced by endothelial cells, promote the recruitment of pericytes to the newly formed blood vessel, what contributes to stabilize and mature the vascular network [44].

Physical and molecular interactions between endothelial cells and their surrounding ECM are also well known. In this context, integrins mediate adhesion to ECM and proteins in order to provide survival cues and traction for invading endothelial cells [45] (Figure 1). Moreover, among other proteases, MMPs play a significant role in modulating angiogenesis by proteolytically remodeling the basement membrane or by exposing chemotactic cryptic motifs sites in the ECM [46]. Chemokines can activate endothelial G-protein-coupled chemokine receptors or recruit pro-angiogenic immune cells [47].

### 2.3. Role of Angiogenesis in Urologic Cancers

**Figure 2:**
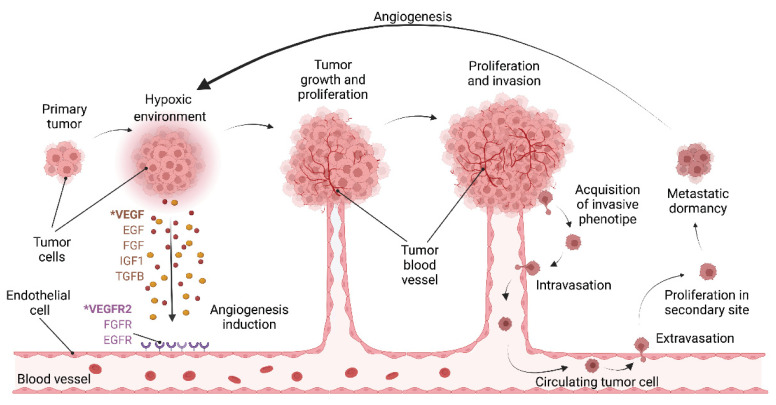
Tumor angiogenesis. Hypoxia within the tumor induces the release of different pro-angiogenic factors, such as VEGFs, EGF, FGF, IGF1 or TGFB1. VEGF-A is the major angiogenic activator and it induces angiogenesis upon binding to VEGFR2, mainly expressed by tumor endothelial cells. The new blood vessels allow exchange of oxygen, nutrients and waste products, leading to tumor growth and proliferation. Moreover, once cancer cells acquire a more invasive phenotype, they can intravasate into blood vessels and reach distant locations leading to metastasis. Disseminated tumor cells that have spread to a secondary site can enter a state of metastatic dormancy or induce angiogenesis and start proliferating.

Although tumor initialization does not entirely rely on angiogenesis, the formation of new blood vessels is required to ensure the supply of oxygen, growth factors and nutrients once the tumor is bigger than few millimeters [48]. In the tumor microenvironment, hypoxia induces the release of VEGFs and other angiogenic factors, activating the angiogenesis switch and favoring tumor growth and intravasation of cancer cells into the blood vessels to reach secondary locations (Figure 2). Thus, angiogenesis is considered one of the hallmarks of cancer as it is needed for tumor growth and cancer cell dissemination to distant organs [49]. Interestingly, upregulated VEGF-A expression levels are detected in most human tumors and is correlated with poor prognosis [50,51,52]. Interaction of VEGF-A and VEGFR2 promotes the increased activity of endothelial cells by inducing proliferation, sprouting, migration, and subsequently the formation of new blood vessels in the tumor microenvironment. It has been already established the importance of angiogenesis in genitourinary cancer survival, progression, and metastasis [53,54,55], and higher microvessel density (MVD) has been associated with worse prognosis in these type of tumors [56,57].

In prostate cancer, the crosstalk between tumor and stromal cells present in the tumor microenvironment is critical for tumor progression and development of distant metastasis [58]. Several endogenous inducers and inhibitors of angiogenesis have already been described to determine the microvascular architecture in these tumors. Accordingly, VEGF-A has been one of the factors extensively studied and it has been found to play an important modulatory role in prostate cancer angiogenesis and metastasis [59]. VEGF-A is overexpressed in prostate tumors and increased in plasma of prostate tumor patients, being the formation of new blood vessel required for prostate cancer metastasis [60] and associated with poor prognosis [61]. Additionally, VEGF-A expression in locally invasive prostate cancer is a prognostic factor for radiotherapy outcome [62]. In the last 3 decades, MVD has been considered a well-stablished marker of tumor progression and metastasis in prostate cancer [63,64], being increased in patients with higher tumor grades, advanced stages and worse outcomes [55]. Currently, MVD predicts survival in prostate cancer patients subjected to active surveillance [54].

VEGF-A and VEGFR2 expression in prostate cancer are regulated by many factors present in the tumor microenvironment, including acetyl-L-carnitine, which downregulates the VEGF-A and other angiogenic pathways in prostate cancer cells [65]. On the contrary, low oxygen and elevated androgens levels increase VEGF-A expression, revealing novel interactions between the androgen receptor, epigenetic and zinc-finger transcription factors and the hypoxia factor HIF-1α [66]. It has been also shown how MMP-3 silencing in prostate cancer cell lines decreases in vitro growth and diminishes Akt and ERK phosphorylation and total VEGFR1 and FGFR3 protein levels. Moreover, in vivo MMP-3 silencing reduces tumor growth and blood vessel formation [67]. Other angiogenesis-related factors with an impact on prostate cancer are miR-185, since it inhibits prostate cancer angiogenesis induced by the nodal/ALK4 pathway and suppress in vivo tumor development [68], and SOCS6, which operates as a tumor suppressor by promoting apoptosis and blocking angiogenesis in prostate cancer [69].

In the metastatic RCC, VEGF-A overproduction has been detected due to the mutation/inactivation of the von Hippel–Lindau tumor suppressor gene. The truncal event in 90% of RCCs consists on the biallelic loss of the Von Hippel–Lindau (VHL) gene, which acts as a negative regulator of the transcription factors HIF1α/2α. Thus, HIF accumulation triggers the hypoxic response in cells and increases angiogenesis [70]. Therefore, indirect HIF inhibitors such as inhibitors of mammalian target of rapamycin (mTOR) (i.e., everolimus and temsirolimus) have shown to be effective in the treatment of RCC [71,72]. On the other hand, pharmacological elimination of VEGFR1^+^ cells may lead the recovery of immunocompetence in metastatic RCC patients and could have a significant impact on the therapeutic efficacy of cancer vaccines or other immune-based therapies [73]. Recently, low miR-125a-3p levels, an inhibitor of the VEGF-A expression, have correlated with poor survival of patients with RCC [74]. The treatment landscape of RCC has been transformed with the advent of antiangiogenic therapies, especially those with tyrosine kinase inhibitors targeting VEGFR2, and immune checkpoint inhibitors [75,76]. Of great interest is the fact that patients with RCC benefit from both treatment options and have shown improved outcomes [77,78]. However, vessel co-option in RCC has been observed as an important resistance mechanism to antiangiogenic therapy [79].

Bladder cancer is one of the most common vascularized cancers [80], and different angiogenesis modulators, such as angiogenin, angiostatin, VEGF-A, HIF-1 and MMPs are important urinary markers in this type of urologic cancer. Similarly to what has been mentioned for prostate cancer and RCC, abnormal VEGF-A expression can be used as a prognostic marker in bladder cancer as well [81], since VEGF-A expression is higher in deeper and invasive tumors than in superficial and non-invasive ones [82]. Furthermore, increased HIF-1α and MMPs expression positively correlates with focal macrophage infiltration, angiogenesis, unfavorable prognosis, recurrence and poor overall survival in urothelial carcinoma [83,84,85]. Of interest, whole-genome sequencing has identified ADGRG6 enhancer mutations and FRS2 duplications as angiogenesis-related drivers in bladder cancer. Functional assays have demonstrated that depletion of ADGRG6 or FRS2 expression in bladder cancer cells compromises their abilities to recruit endothelial cells and induce tube formation. Moreover, HRAS/KRAS, PI3K, FGFR1/FGFR3, FAK, mTOR and PKCB/PKCG, genes with important roles in angiogenesis, were altered in 23%, 22%, 17%, 8%, 7% and 7% of the bladder tumors, respectively [86]. Among bladder cancers, urothelial cancer and non-muscle-invasive bladder cancers could benefit from anti-FGF therapy. FGFR alterations, especially in FGFR3, are much more common in non-muscle-invasive bladder cancers and tumors harboring FGFR3 alterations are potentially vulnerable to FGFR3-targeted therapies [87,88,89].

In testicular tumors, especially those originated in the malignant germ cells, the production of proangiogenic molecules induces the vascular and lymphatic invasion and represents a well-known feature associated with metastatic progression [90]. In agreement, VEGF-A, VEGFR2 and thrombospondin expression is correlated with MVD and increased in testicular tumors [91,92,93]. Moreover, MMP-2 and MMP-9 are upregulated in experimental teratocarcinoma models and in embryonal cell carcinoma, correlating to greater invasiveness [90].

As discussed in the next section of this manuscript, several antiangiogenic agents have shown to efficiently inhibit urologic tumor growth and reduce metastatic invasion. However, as previously discussed and summarized in Figure 1, the different angiogenic stimulators share overlapping activities and signaling cascades, which can explain that antiangiogenic therapies based on inhibitors interfering with only a single angiogenesis activator could finally promote the appearance of resistance or suboptimal effectiveness [94,95].

## 3. Antiangiogenic Therapies of Cancer

### 3.1. Use of Inhibitors of Angiogenesis in Cancer

As already mentioned, it is now widely accepted that a deregulated and persistent activation of the angiogenic switch is one of the hallmarks of cancer [49,96]. The hypothesis that a continuous blood supply is needed for tumor growth and consequently tumor dormancy could be maintained by preventing neovascularization of microscopic cancers, was pioneered about 50 years ago by Judah Folkman [97]. Presumably, this antitumor strategy should present a number of advantages over traditional chemotherapy. By targeting activated endothelial cells, which are responsible for the formation of new blood vessels, it should be applicable to a wide variety of tumors. Moreover, the low mutagenic potential of endothelial cells predicted a lower occurrence of resistance to the therapy than in the case of traditional chemotherapy, targeting tumor cells [34,98].

**Figure 3:**
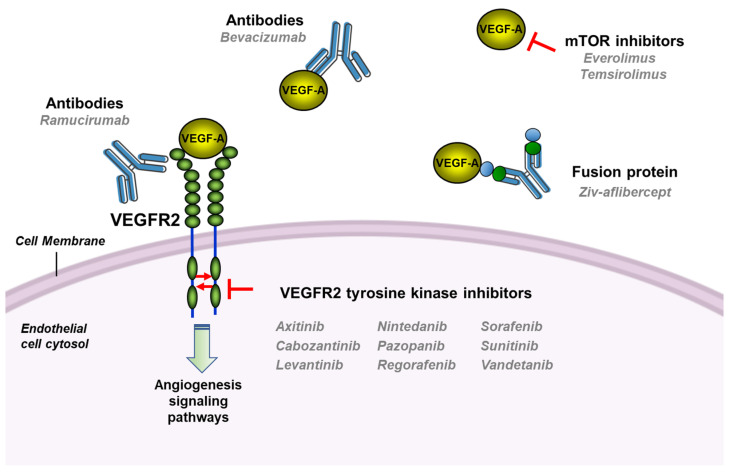
Main molecular targets for the antiangiogenic drugs approved in oncology.

Despite the enthusiasm aroused by this therapeutic strategy, it was not until 2004 when the first antiangiogenic drug received the US Food and Drug Administration (FDA) approval for the treatment of cancer patients [98,99]. Since then, the number of angiogenesis inhibitors approved for clinical use has been growing steadily, so that there is now an armamentarium of antiangiogenic drugs that can be used for the treatment of cancer patients (Figure 3 and Table 1). The body of clinical evidence confirming their success in an increasing number of cancers reaffirms that angiogenesis inhibition is one of the most promising antitumor targets [34,100,101].

The pivotal role played by VEGF-A in activating tumor angiogenesis prompted that the majority of antiangiogenic therapies were designed to neutralize the activation of endothelial cells by this angiogenic factor, either by directly blocking VEGF-A or by inhibiting the activation of VEGFR2 [36] (Figure 3). Probably the most successful antiangiogenic drug in the clinic is bevacizumab, a humanized neutralizing antibody that blocks VEGF-A, preventing its binding to the receptor VEGFR2. Since its first approval in 2004 to be used in combination with fluorouracil-based chemotherapy as a first-line treatment for metastatic cancer of the colon or rectum, bevacizumab indications have extended to many other types of tumors, including non-small-cell lung cancer, breast cancer, glioblastoma or metastatic renal cell carcinoma, among others, but always in combination with standard chemotherapy [102,103]. VEGF-A can also be neutralized by Ziv-aflibercept (VEGF-Trap), which combines ligand-binding elements taken from the extracellular components of VEGF receptors, fused to the Fc portion of IgG. This soluble fusion protein has been recently approved for the treatment of metastatic colorectal cancer and neovascular age-related macular degeneration [104,105,106].

A continuously increasing number of low molecular weight drugs have been developed to inhibit the VEGFR2 activation (Figure 3 and Table 1). Sorafenib, an inhibitor of the VEGFR2 tyrosine kinase domain, received the FDA approval in 2005 for the treatment of RCC, based on the results obtained in two clinical trials in which the progression-free survival was significantly improved in patients treated with this drug [107]. Interestingly, this compound is also an inhibitor of the Raf/MEK/ERK and the PDGFR signaling pathways. Almost at that same time, the multitargeted inhibitor sunitinib, which inhibits VEGFR2, PDGFR-β, FLT-3 and the stem cell factor receptor Kit, received FDA approval for patients with gastrointestinal stromal tumors and advanced kidney cancer, this being the first time that this agency had approved a new oncology product for two indications simultaneously [108].

Since then, a number of molecules have been expanding the group of tyrosine kinase inhibitors (TKI) capable of inhibiting the VEGFR2 activation. They are characterized by inhibiting not only VEGF-A pathway, but also those of other factors involved in angiogenesis regulation. The observation that these multikinase inhibitors show activity as monotherapy reinforces the idea that multitargeted approaches may be needed to reach an effective inhibition of tumor angiogenesis [34,109,110].

Concerning urologic tumors, the greatest benefits have been achieved in kidney tumors patients, so that the development of antiangiogenic therapies marked the beginning of a golden age in the treatment of metastatic RCC. As mentioned before, inactivation of the tumor suppressor VHL is a feature of the most common and aggressive malignant subtype of RCC [111]. The aberrant accumulation of HIF proteins, derived from the VHL loss, results in the uncontrolled activation of the angiogenic switch, what makes this type of tumors good candidates for antiangiogenic therapies. Indeed, advances in the treatment translated into significant increases in survival after the introduction of anti-VEGF therapies for RCC patients [76,112]. The approvals of sorafenib and sunitinib were followed in successive years by those of other anti-VEGF therapies, including the combined use of bevacizumab and interferon alpha, or the development of other VEGFR2 inhibitors such as pazopanib, axitinib, cabozantinib and levantinib, which presented a higher objective response, less cytotoxicity or better quality of live than earlier treatments [5,76].

The fact that mutations in phosphatidylinositol-3 kinase, a kinase upstream of mTOR, are also common in metastatic RCC is the rationale for the use of therapies that target this pathway [113]. Interestingly, mTOR signaling pathway is often activated in advanced RCC, what contributes to promote several processes involved in tumor angiogenesis, including the production of pro-angiogenic factors, such as VEGF-A [114]. The mTOR inhibitors temsirolimus and everolimus have been approved by the FDA as single agents in the second line setting and in the first line in RCC patients who were in the poor prognosis category [115,116].

The crucial role played by angiogenesis in prostate cancer fueled the development of antiangiogenic strategies in the clinical setting. They included phase II and III trials using most of the anti-VEGF inhibitors shown in Table 1 (bevacizumab, aflibercept and several VEGFR2 TKIs) on localized and hormone-sensitive disease, or castration-resistant refractory disease (recently reviewed in [117,118]). Nevertheless, results derived from those clinical trials have been disappointing so far, with many of them demonstrating increased toxicity with no clinical benefit when added to chemotherapy or hormonal therapy [59].

The results of several clinical trials with angiogenesis inhibitors in bladder cancer were equally frustrating. Based on the observation that angiogenesis is relevant to the progression of this type of tumor, several VEGFR2 TKIs have been investigated in phase II trials, either as monotherapy or in combination with standard chemotherapy. These studies were interrupted due to the lack of efficacy or increased toxicities [119,120]. However, targeting angiogenesis via either the VEGF-A or mTOR pathways has demonstrated activity in a small number of urothelial carcinoma patients [121,122]. Finally, FGF pathways are also being explored in bladder cancer treatment. In this regard, erdafitinib, a pan-FGFR kinase inhibitor has been recently approved by the FDA for patients with locally advanced cancer or metastatic urothelial carcinoma with certain FGFR3 or FGFR2 genetic alterations and that had progressed during or following platinum-based chemotherapy [123,124].

From the above exposed it can be concluded that not everything has been successful in the clinical development of antiangiogenic therapies. A limitation of anti-VEGF therapeutic strategies is the need of using them in combination with other therapies. A possible explanation is that anti-VEFG drugs can “normalize” the aberrant tumor vasculature, so that it would become more functional, allowing “traditional” chemotherapy to reach the tumor and function more effectively [125,126]. In this line, the use of antiangiogenic strategies is currently being explored to increase the efficacy of immunotherapy, an important therapeutic modality that is revolutionizing cancer treatment [127,128,129]. In addition, the limited clinical success achieved by some antiangiogenic monotherapies could be explained by the great complexity of the regulation of angiogenesis, exerted by a complex network of pro- and antiangiogenic factors [33]. This complexity may also be the reason for the appearance of resistance to antiangiogenic treatments by a number of mechanisms that include upregulation of alternative proangiogenic signals, increased production of proangiogenic factors by stromal cells, activation of an invasive phenotype, or induction of an alternative mechanism of vascularization [95,130,131]. Hence, it seems that a greater degree of regression and more durable responses would be expected if multi-targeted therapies, able to block multiple angiogenic pathways simultaneously, were used [109,132].

### 3.2. Angioprevention

The idea of using angiogenesis inhibitors as chemopreventive agents, capable of arresting both early primary tumor growth and metastasis, was inherent to Folkman’s initial proposal. It was reformulated three decades later by Adriana Albini’s group, who coined the term “angioprevention”, understood as the prevention of cancer by inhibition of tumor angiogenesis [133]. These authors observed that many chemopreventive molecules were also inhibitors of angiogenesis, and came to the conclusion that many of the antiangiogenic drugs designed for anticancer chemotherapy could be useful for cancer prevention [134]. According to this reasoning, dietary inhibitors of angiogenesis could be potential drug candidates for the angioprevention of cancer.

**Figure 4:**
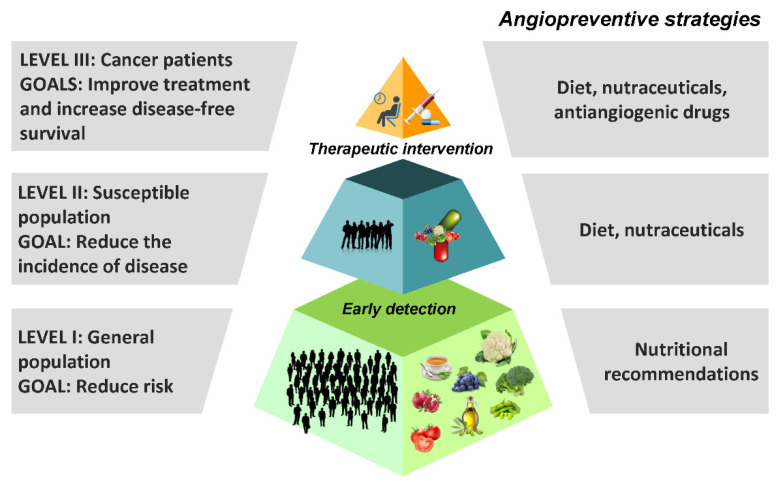
Different angiopreventive strategies can be implemented, depending on the targeted population.

Angioprevention can be implemented at any of the three levels of chemoprevention, depending on the targeted population (Figure 4) [135]. Angiogenesis inhibition may contribute to slow down the progression of the tumor at very different stages of the malignization process. Besides avoiding the activation of the angiogenic switch in some predisposing conditions, including chronic inflammation, pre-neoplastic or hyperplastic lesions, it may help the host defense systems to more efficiently avoid the development of clinically detectable tumors [136].

As a result of primary angioprevention initiatives, the incidence of disease could be reduced in a broad healthy population by means of nutritional recommendations, dietary supplements, and natural angiogenesis inhibitors that could help to stop early events in tumorigenesis. Indeed, diet and nutrition underlie many of the large international differences in incidence seen for most cancers [137,138].

At a second level of angioprevention, population at high-risk for developing cancer due to genetic abnormalities, usually associated with their lifestyle, could benefit of the inhibitory effect of antiangiogenic compounds on the growth of undetectable primary tumors. Vegetables, fruits and medicinal herbs are a valuable source of inhibitors of angiogenesis that could be used as chemopreventive agents. The consumption of these compounds, either as nutraceuticals or being part of a food that provide medical and health benefits, should be less expensive, safer and more available than that of synthetic antiangiogenic drugs. This makes them particularly suitable for earlier preventive strategies and facilitates their widespread use in the long term schedules required for an effective chemoprevention of cancer [138,139].

Finally, tertiary angioprevention could be based on the use of any of the inhibitors of angiogenesis that have been approved for their clinical use, administered either throughout or after the treatment in order to prevent a relapse. More sustainable and less aggressive antiangiogenic strategies, as those used for primary and secondary prevention, could also be desirable in order to halt the growth of those undetectable microscopic metastasis that could remain after primary tumor resection in cancer patients [140,141,142].

## 4. Dietary Phytochemicals in Angioprevention of Urologic Cancers

Although specialized diets are not the only way to decrease the chances to develop cancer, those that are enriched in antiangiogenic molecules could be used to prevent the activation of the angiogenic switch in the early steps of tumor progression as well as in the micrometastasis awakening from their dormant state. Among the different strategies for cancer prevention, dietary and nutritional interventions have been widely explored in urological cancers. In many cases, they have been based on foods that are rich in phytochemicals, which are believed to be responsible for their protective or disease preventive properties. In general, phytochemicals are secondary metabolites that are synthesized in response to the interactions of the plant with the environment (as a defense system) or as a part of the reproductive mechanism of the plant (for example to attract insects for the promotion of pollination). Since phytochemicals are found in fruits, vegetables, beans and grains, they can be easily incorporated to a balanced diet rich in plant-derived foods, what could also ensure a continuous supply of a combination of these beneficial compounds. In addition, nutritional advice aimed at increasing the proportion of fresh vegetables in the average person’s diet is generally well received by the general population, which is increasingly willing to adopt healthier lifestyles.

Many plant-derived foods and their health-promoting phytochemicals are being explored in the prevention of diseases, including urologic cancer. Although their effects have been mainly studied in prostate cancer, sometimes their action have also been extended to other types of tumors. In this section, epidemiological evidence supporting the role of certain foods in urologic cancer prevention will be presented, with particular emphasis on their content in bioactive compounds that could be used in the angioprevention of cancer.

### 4.1. Vegetables

#### 4.1.1. Cruciferous Vegetables

Vegetables belonging to the family *Brassicaceae* (previously named *Cruciferae*), including broccoli, cauliflower, cabbage, kale and Brussels sprouts, among others, are widely consumed around the world. Their popularity has increased in the last years due to the finding that they are rich in phytochemicals that may be used in the prevention or treatment of chronic diseases such as obesity, cardiovascular diseases (hypertension, stroke), cancer, type-2 diabetes and osteoporosis [143].

**Figure 5:**
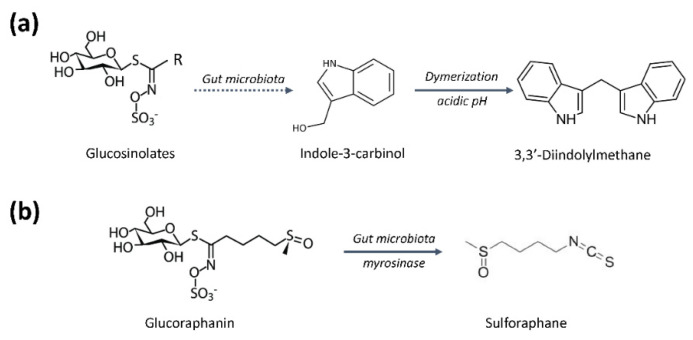
Role of gut microbiota in the production of active phytochemicals from cruciferous vegetables. (**a**) Indole-3-carbinol and 3,3′-diindolylmethane are generated from inert glucosinolates after digestion. (**b**) Glucoraphanin, a glucosinolate found almost exclusively in broccoli is converted into the chemopreventive compound sulforaphane through enzymatic catalysis by plant myrosinase or β-thioglucosidases in the gut microflora.

A high cruciferous vegetable consumption has been associated in several human studies with a lower risk of several types of cancer, what suggests that it could be a possible cost-effective approach to cancer prevention through dietary intervention. The anticancer properties of these foods are attributed to bioactive indoles and isothiocyanates, such as indole-3-carbinol and sulforaphane, respectively, generated in the digestive system from general precursors called glucosinolates (Figure 5) [144,145]. Similar to other examples of dietary intervention for cancer prevention, there are conflicting data in the literature regarding the efficacy of consumption of *brassica* vegetables in reducing cancer risk. Several reasons for the observed discrepancies include changes in the phytochemical content in vegetables due to growing conditions and cultivar differences, or the ways in which they are prepared and cooked [146]. This may have driven a transition from fresh sprouts to sprout extracts, to powders, capsules and dietary supplements made from them, in order to make more reproducible the delivered dose of the desired phytochemical [147,148,149]. Differences in the pharmacokinetic properties of glucosinolates between and within the populations studied should also be kept in mind. In this sense, the gut microbiome may contribute to the observed inter-individual variations [145,150].

Diets high in cruciferous vegetables have been correlated with a lower risk of incidence and aggressiveness of prostate cancer in several case-control studies [151,152,153,154]. This is in agreement with results from some cohort studies, although it could not be confirmed by others [155,156,157,158,159]. A significantly decreased prostate cancer risk was observed overall in the cruciferous vegetables’ intake group in a meta-analysis. Moreover, results from the first clinical trial of sulforaphane-rich extracts in men with prostate cancer revealed a positive effect in decreasing PSA levels, in spite of having not achieved its primary endpoint [153,154].

There is also a large body of evidence supporting the beneficial role of cruciferous vegetables in bladder cancer. A large prospective cohort epidemiologic study revealed that intake of cruciferous vegetables, particularly broccoli, had a strong inverse association with bladder cancer risk [160]. These results were in agreement with others from two retrospective case-control studies, that also indicated that genetic variants of the consumers and the way these vegetables were eaten, either raw or cooked, could modify this association [161,162]. In this regard, a cohort study found a significant inverse association of bladder cancer mortality and raw broccoli intake [163]. In another case–control study, the protective effect of dietary isothiocyanates was even most evident in older individuals and heavy-smokers [164]. Several meta-analysis have found a significant reduction in the bladder cancer risk associated to an increased cruciferous vegetables intake [165,166,167,168]. Interestingly, in a Multiethnic Cohort Study, the protective effect of fruits and vegetables against bladder cancer was found to be more pronounced in women than in men [169]. On the contrary, some other trials and studies have failed to demonstrate a relationship between the consumption of these vegetables and the risk of bladder cancer [167,170]. Among them, findings from a recently published study, including over 500,000 participants from diverse areas across the United States, showed no relationship between cruciferous vegetable intake and bladder cancer [171]. Intervention studies have shown the feasibility of implementing dietary modifications in bladder cancer patients aimed on preventing incident, recurrent, and progressive disease [172]. Overall, in spite of evidence supporting the notion of potential beneficial roles of a diet rich in cruciferous vegetables in bladder cancer prevention, better designed prospective studies are also needed in this field in order to fully demonstrate this beneficial effect.

Finally, several case control studies have shown that there is an inverse association between cruciferous vegetables intake and RCC [173,174]. The preventive role of these vegetables in RCC has also been supported by the findings of some meta-analyses [175,176].

As mentioned before, cruciferous vegetables are a rich source of glucosinolates, which can generate the chemopreventive phytochemicals (Figure 5). The content of glucosinolates depends on the cruciferous variety and is determined by the place and the cultivation method. There is evidence that very little content of intact glucosinolates is absorbed, being their hydrolyzed products (typically isothiocyanates and indoles) those that can be absorbed (Figure 5a). In this regard, chopping and chewing break the plant structure and promotes the hydrolyzation of glucosinolates through the catalytic actions of plant myrosinase or β-thioglucosidases in the gut microbiota [147]. The cooking temperature can inhibit the activity of this enzyme and consequently limit the formation of bioactive phytochemicals, including sulforaphane, indol-3-carbinol and 3,3′-diindolymethane [177].

Indole-3-carbinol and 3,3′-diindolymethane suppress angiogenesis in vivo and in vitro, being the later the one showing the strongest antiangiogenic activity. Both compounds are able to inactivate the ERK1/2 pathway, but they differ in their potential to regulate the main endothelial survival signals, caspases activation and Akt pathway inactivation, which are only affected by 3,3′-diindolymethane. Indole-3-carbinol suppresses tumor-induced angiogenesis in a mouse dorsal air sac assay, possibly through the inhibition of tube formation and induction of apoptosis in endothelial cells [178], and 3,3′-diindolymethane reduces neovascularization in the in vivo Matrigel plug angiogenesis assay and inhibits up to 64% the growth of human MCF-7 cell tumor xenografts in female athymic (nu/nu) mice [179].

Glucoraphanin is a glucosinolate found almost exclusively in broccoli. In the digestive system is converted into sulforaphane, which is believed to be the compound responsible for many of the health benefits attributed to this vegetable (Figure 5b). Consideration of broccoli as a “super vegetable” is in the origin of the launch of a “super” broccoli in U.K. supermarkets, a hybrid strain containing up to three times higher levels of glucoraphanin than that of normal broccoli strains [180]. More recently, it has been reported that enhancing glucoraphanin content in broccoli by genetic engineering results in enhanced exposure of human tissues to sulforaphane in a manner that might provide health benefits [181]. Sulforaphane affects in vitro angiogenesis through the regulation of the FOXO transcription factor and the inhibition of the MEK/ERK and PI3K/Akt pathways [182]. In addition, sulforaphane suppresses HepG2-stimulated HUVEC migration, adhesion and tube formation, most likely through its interference with the STAT3/HIF-1α/VEGF-A signaling cascades in HepG2 cells. A significant reduction has also been seen in HepG2 tumor growth in a modified chicken egg chorioallantoic membrane (CAM) assay, probably associated with a decrease in HIF-1α and VEGF-A expression within tumors [183]. And lately, it has been reported that daily administration of sulforaphane (100 nmol/day, i.v. for 7 days) to female Balb/c mice bearing VEGF-A-embedded Matrigel plugs reduces angiogenesis progression measured by hemoglobin concentration [184].

**Figure 6:**
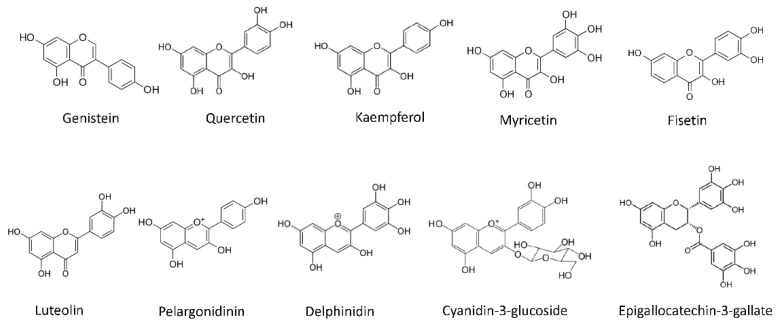
Chemical structures of some flavonoids found in vegetable and fruits, including isoflavones (genistein), flavonols (quercetin, kaempferol, myricetin and fisetin), flavones (luteolin), anthocyanidins (pelargonidin, delphinidin and cyanidin-3-glucoside) and flavan-3-ols (epigallocatechin-3 gallate).

Kaempferol (Figure 6) is an antioxidant flavonol found in many fruits and vegetables. The richest plant sources of kaempferol are kale (*Brassica oleracea*), Chinese cabbage (*Brassica rapa*), broccoli, spinach and herbs such as dill, chives and tarragon [185]. Many studies have described the beneficial effects of dietary kaempferol in reducing the risk of chronic diseases, especially in cancer. Actually, epidemiological studies have shown an inverse relationship between kaempferol intake and cancer [186]. Kaempferol impairs angiogenesis and tumor angiogenesis both in vitro and in vivo by inhibiting VEGF-A expression and secretion [186,187]. Its effects have been already observed in different angiogenesis models, and it seems to have an important role HIF-1α, VEGFR2 and its downstream signaling cascades, such as Akt, mTOR and MEK1/2–ERK1/2 [188,189,190].

#### 4.1.2. Soy

Soy (*Glycine max)* is a plant that belongs to the family of the *papilionaceous*. Soy foods consumption has been associated with prevention of cardiovascular diseases and some types of cancer. In addition, it may help in managing depression, hypercholesterinemia, and easing menopausal symptoms, among other beneficial effects [191].

Prostate cancer incidence is lower in Asian countries where soyfoods are commonly consumed as compared to that of Western countries. In addition, results from several case-control studies indicate that a higher soy consumption by Asian men is associated with as much as a 50% reduction in prostate cancer risk. Several epidemiologic studies associate a reduced risk of prostate cancer with higher consumption of soy and nonfermented soy foods [192]. Some data from phase I-II randomized clinical studies suggest a preventive effect of isoflavones and soy products, typically demonstrated by a decrease in serum PSA or PSA-doubling time [193,194,195,196,197,198,199]. Nevertheless, other studies have not been able to confirm these results [200]. The idea that the combination of phytochemicals could provide synergies in their preventive activity emerges from the observation that after six months of treatment, a combination of curcumin and soy isoflavones, was more effective in lowering PSA levels than any of them individually [201]. Although clinical studies agree that soy products are generally well tolerated by patients, their potential in cancer prevention and treatment have not been consistent enough in many cases, often due to the limited size of samples and short duration of the studies [202].

Genistein (Figure 6), originally labeled as a phytoestrogen, is one of the major isoflavones found in soy products and has been shown to inhibit cancer growth in vitro and in vivo [194,203]. It is a multitargeted antitumoral drug displaying effects on cell cycle, apoptosis, angiogenesis, invasion and metastasis, probably mediated by the inhibition of Akt, NFκB, MMPs and Bax/Bcl-2 signaling pathways [194,203]. In vitro, genistein inhibits endothelial cell proliferation at concentrations that are in the range of those found in urine of subjects consuming a plant-based diet. Moreover, genistein downregulates the expression of several molecules responsible for the control of angiogenesis, such as VEGF, PDGF and MMPs, and upregulates that of angiogenesis inhibitors, including plasminogen activator inhibitor-1, endostatin, angiostatin and thrombospondin-1 [204,205,206]. The antiangiogenic activity of genistein is mediated by the inhibition of HIF pathway in pancreatic carcinoma cells [207] and by down-regulation of the pro-angiogenic factors expression via inhibiting protein tyrosine kinase activity both in breast cancer cells and in xenograft tumors. The inhibition of this activity and that of MAPK pathway interrupt VEGF-A-stimulated endothelial cell activation [208]. This suggests that genistein could contribute to the cancer preventive effect of a plant-based diet, by inhibiting neovascularization [209].

### 4.2. Fruits

#### 4.2.1. Tomato

Tomato, the fruit of a plant of the *Solanaceae* family, is included among the top five most popular fresh-market vegetables. With the discovery of the New World, it became part of the Italian and Spanish diet between the 16th and 17th centuries, where it was mainly consumed raw, seasoned with oil, salt and pepper. It was not until the 18th century that tomatoes began to be used as a sauce, what contributed to making it one of the most widely consumed foods in the world. Currently it is cultivated and consumed all over the world and, despite its seasonality linked to summer, cultivation in greenhouses and canning allows it to be available all year round. Its low caloric content, interesting nutritional value, in addition to prominent antioxidant, anti-inflammatory, cardioprotective and anticancer activities, make tomato an excellent ally in healthy cooking and a useful ingredient for the development of functional foods [210,211,212].

**Figure 7:**
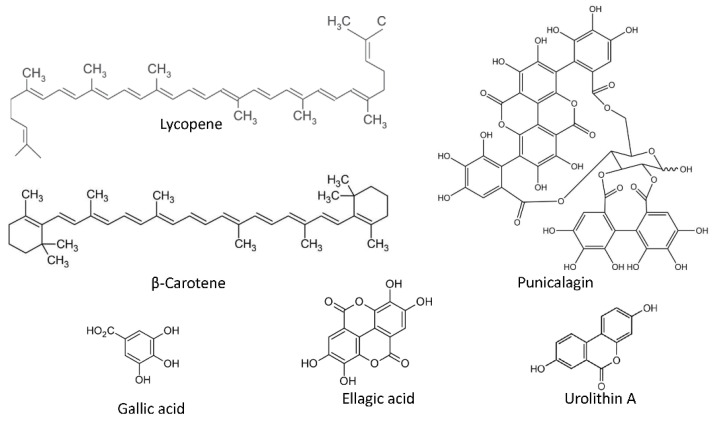
Chemical structures of some antiangiogenic phytochemicals found in fruits. They include carotenoids (lycopene and β-carotene), rich in tomato, punicalagin, the major fruit ellagitannin, abundant in pomegranate and other compounds derived from the hydrolysis of gallitannins and ellagitannins (gallic acid, ellagic acid and urolithin A).

Rich in micronutrients as vitamin C, potassium and folic acid, ripe tomato fruits also contain a high variety of carotenoids and polyphenols in different concentrations, most of them considered as chemopreventive compounds [213]. Carotenoids are C40 tetraterpenoid pigmented molecules abundant in multiple fruits and vegetables. Among the carotenoids present in ripe tomato, lycopene (Figure 7) is the most abundant and may be responsible for many of the health promoting effects of this fruit. More than 80% of dietary lycopene derives from tomato or tomato-based products, including juice, soup, pizza and sauces. Other sources include watermelon, pink grapefruit and papaya [214]. A large number of epidemiological studies indicate that daily intake of 2−20 mg lycopene has significant benefits in the prevention and treatments of cardiovascular diseases, neurodegenerative disorders, and several types of cancers [215].

Lycopene can exert its anticancer effects through various mechanisms, including apoptosis induction, cell motility inhibition, adhesion and migration, decrease in inflammatory cytokines, decrease in PSA serum level and angiogenesis inhibition [216]. Moreover, this carotenoid exhibits a remarkable in vitro and in vivo antiangiogenic activity [217,218,219], as it is associated with a reduction of the angiogenic markers in prostate tumors [220]. The antiangiogenic activity shown by this carotenoid reinforce the role of lycopene in angioprevention. Its main mechanism of action involves the inhibition of the MMP-2/plasminogen activator (uPA) system through VEGFR2-mediated PI3K-Akt and ERK/p38 signaling pathways in endothelial cells [221], although an immunomodulatory role of this compound has also been described in human mononuclear cells [221].

Regarding urologic cancers, several epidemiologic studies have reported that consumption of tomatoes and tomato products is associated with a reduced risk of prostate cancer [222,223,224,225]. Clinical trials utilizing lycopene in prostate cancer patients with different stages of disease have yielded some promising results. In these clinical trials doses from 10 to 120 mg/d were well tolerated, with very occasional gastrointestinal toxicities. Some studies have reported a correlation between low concentrations of lycopene, either in blood or in prostate, and a lower risk of prostate cancer [226,227,228,229,230,231]. All these observations have enabled several companies to obtain a designation of Generally Recognized as Safe (GRAS) from the U.S. FDA for their lycopene-containing products. Chemopreventive role of lycopene in other types of urologic cancers cannot be discarded. Accordingly, an increase in lycopene intake among postmenopausal women could be correlated with a lower risk of RCC [232]. However, in spite of all the above-mentioned promising results, further large-scale randomized trials are needed to fully determine the role of lycopene in chemoprevention of cancer [233].

Other phytochemical found in tomato is β-carotene (Figure 7), which belongs to the carotenoids group and is present in ripe tomatoes, as well in many other fruits and vegetables. Its antiangiogenic activity has been described in vitro, ex vivo and in vivo. In endothelial cells, β-carotene is able to reduce cell growth, migration and tubular-like structures formation on Matrigel, downregulating the expression of extracellular matrix enzymes such as MMP-2, MMP-9, prolyl-hydroxylase and lysyl-oxidase, and upregulating the expression of tissue inhibitors of metalloproteinases TIMP-1 and TIMP-2 [234].

#### 4.2.2. Pomegranate

Pomegranate (*Punica granatum*) is a perennial plant with wide applications in traditional medicines. It has been associated to favorable health benefits including the control of obesity and diabetes, and antioxidant, anticancer and anti-inflammatory properties [235].

Several studies have examined the effect of interventions with pomegranate products in prostate cancer patients, showing that pomegranate juice administration (8 oz per day for up to 33 months) is well tolerated. In a Phase II placebo-controlled study, daily consumption of pomegranate juice for several months induced a noteworthy extension of the PSA-doubling time in men with rising PSA following radical prostatectomy or radiotherapy [236]. These early results were confirmed not only in patients with rising PSA following initial therapy, but also in subjects with clinically localized prostate cancer undergoing active surveillance [237,238]. This is in agreement with the results from a randomized controlled trial, testing the effect of an extract of polyphenol-rich whole food supplement in men with localized prostate cancer. In those men that received for six months an oral capsule containing a blend of pomegranate, green tea, broccoli and turmeric, the PSA levels reduced to 14.7%, in contrast to 78.5% of those men in the placebo group [239]. Although several companies distribute pomegranate as a dietary supplement, FDA has not yet approved it for cancer treatment or prevention. Further randomized placebo-controlled studies are needed to elucidate the chemopreventive potential of pomegranate and its products.

The peel and fruit of pomegranates and walnuts are rich in ellagitannins, being punicalagin (Figure 7) the most abundant ellagitannin in pomegranate [240]. These phytochemicals are readily metabolized by gut microbiota, generating the active forms ellagic acid and urolithin A derivatives (Figure 7) [241]. Preclinical experiments show that ellagitannins inhibit prostate cancer proliferation and angiogenesis under hypoxic conditions and induce apoptosis [241,242]. According to a tissue distribution experiment in wild-type mice, the prostate gland rapidly takes up high concentrations of urolithin A after oral or intraperitoneal administration (0.3 mg/mouse/dose). Ellagic acid can also be detected in the prostate following intraperitoneal, but not oral, administration of pomegranate extract (0.8 mg/mouse/dose) [243].

Concerning its antiangiogenic properties, ellagitannin-rich pomegranate extracts inhibit endothelial proliferation in both normoxic and hypoxic conditions. In addition, ellagitannin-rich pomegranate extracts reduce the proliferation of androgen dependent human cancer cells (LNCaP) in hypoxia, and incubation of LNCaP cells and HUVECs with 0–5 μg/mL of ellagitannin-rich pomegranate extract for 48 h decreases in a dose-dependent manner the secretion of VEGF-A to the conditioned media. Interestingly, in vivo studies show that tumor volume in SCID mice bearing a human prostate cancer xenograft (LAPC4) that were treated with pomegranate extracts was greatly decreased. In addition, the tumor blood vessel density decreased and VEGF-A plasma levels were lower when compared with control mice [240]. In another study, pomegranate seed oil and fermented juice polyphenols induced a significant decrease in the newly formed blood vessel in the CAM assay [244].

As mentioned above, ellagitannins are metabolized to the active form ellagic acid. Ellagic acid interferes with different in vitro angiogenic steps, including endothelial cell proliferation, migration and tube formation [245], as well as it inhibits the vessel formation in the CAM assay, the endothelial sprouting in chicken aortas and the size of MDA-MB-231 breast cancer xenografts [246]. Mechanistically, ellagic acid inhibits MMP-2 secretion, HIF-1α-induced VEGF-A/VEGFR2 signaling, VEGFR2 tyrosine kinase activity and its downstream MAPK and PI3K/Akt signaling pathways [247,248]. Of note, molecular docking simulation indicates that ellagic acid could form hydrogen bonds and aromatic interactions within the ATP-binding region of the VEGFR2 kinase unit [246].

Punicalagin and gallic acid (Figure 7), present in pomegranates have also shown interesting antiangiogenic properties. On one hand, punicalagin is able to inhibit proliferation and migration, and induce apoptosis in osteosarcoma cells, interfering with osteosarcoma development and tumor angiogenesis in a subcutaneous tumor xenograft model [249]. Furthermore, punicalagin suppresses the vascular network formation in the CAM assay [250]. On the other hand, gallic acid is partially responsible for the in vitro antiangiogenic activities of *Rubus* leaf extract. This phytochemical inhibits angiogenesis, as revealed by the neovessel decrease in a human placental vein model and in rats treated with *Rubus* leaf extract [250,251]. Additionally, gallic acid decreases tube formation in normal brain endothelial cells [251] and in HUVECs incubated in presence of ovarian tumor cells conditioned medium. In the latter, the mechanism regulating the antiangiogenic effect seems to be through the downregulation of VEGF-A and HIF-1α expression in ovarian cancer cells [252]. Although less studied, pelargonidin (Figure 6), an anthocyanin present in pomegranates, also displays promising antiangiogenic properties in zebrafish embryos exposed to this compound [253].

#### 4.2.3. Grapes

Grapes, the fruit of *Vitis vinifera*, are eaten or used to make juice and wine. The grape seeds and skins, a relevant part of the solid residues generated during the winemaking process, are also industrially processed to produce extracts and used as nutraceuticals due to their health benefits [254,255]. Grapes and their products are rich in phytochemicals, mainly polyphenols, which have antioxidant properties and may help to reduce the risk of heart disease, as it has been reported for a moderate intake of red wine or grape seed extract, among other studies [256,257,258]. Red wine polyphenolic compounds also exhibit antitumoral activities, which could be, at least in part, mediated by their antiangiogenic activity [259,260]. Grape seed proanthocyanidins are dietary supplements used for cancer prevention, and their mechanism of action seems to be also linked to their angiopreventive properties [261,262].

Although some epidemiological studies have examined the effect of grapes consumption in relation to the risk of urologic cancers, few consistent results have been achieved. A study of the relation between fruit and vegetable consumption and the risk of bladder cancer in the European Prospective Investigation into Cancer and Nutrition, indicate that a 25 g/day increase in leafy vegetables and grapes consumption was associated with a reduced risk of non-aggressive urothelial cell carcinoma of the bladder [263]. A commercial preparation of pulverized muscadine grape (*Vitis rotundifolia*) skin has been evaluated as a therapeutic option for patients with nonmetastatic biochemically recurrent prostate cancer. In a phase I/II study, patients were assigned to increasing doses of MuscadinePlus (MPX), containing ellagic acid, quercetin and resveratrol, demonstrating that muscadine grape skin extract is safe in a wide range of concentrations [264]. Nevertheless, results from a randomized, multicenter, placebo-controlled and dose-evaluating phase II trial, carried out on 112 biochemically recurrent prostate cancer patients, did not demonstrate a significant shortening of the PSA-doubling time in those patients that received MPX. Exploratory analysis of some results derived from this study revealed a patient population that could have a potential benefit [265].

The list of phytochemicals found in grapes is long, including the flavonols quercetin and kaempferol, ellagic acid and some proanthocyanidins. Some other interesting antiangiogenic compounds found in grapes are resveratrol, piceatannol, fisetin, delphinidin and myricetin.

**Figure 8:**
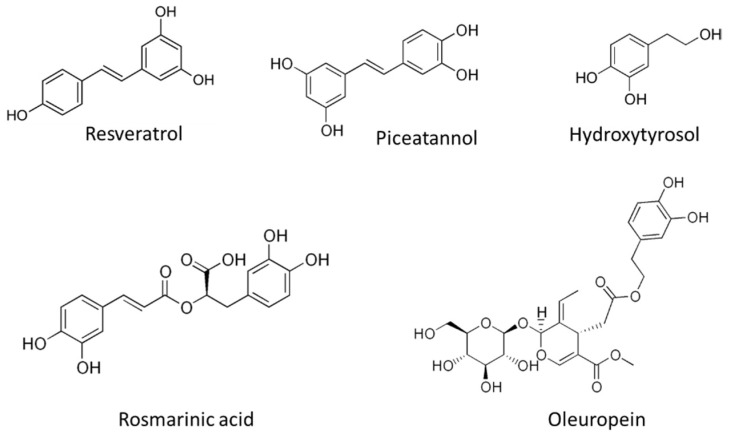
Chemical structures of several polyphenolic compounds found in vegetables, fruits and beverages.

Resveratrol (Figure 8) is one of the most studied polyphenolic compounds in fruits and it is present in the skin of grapes and other fruits (mainly berries), pistachio nuts and peanuts. It has been described to exhibit antioxidant, antidiabetic, antitumor and anti-inflammatory properties. Interestingly, resveratrol displays a clear antiangiogenic activity, reducing VEGF-A expression in tumor cells, and suppressing the endothelial cell response to this angiogenic factor [266,267,268,269]. Although the exact target of this compound is not clearly defined, several mechanisms for its antiangiogenic activity have been already described. The interference of resveratrol in different molecular axes related to VEGF-A/VEGFR2 pathway (such as HIF-1α and GSK3b/β-catenin/TCF), and the inhibition of TFG-β pathway by this natural compound, could be responsible for its modulatory effect [269,270,271]. In addition, the antiangiogenic activity of resveratrol is related with a reduction of aerobic glycolysis activity in VEGF-A-activated endothelial cells, through a mechanism that implicates the interference with the ERK-mediated pyruvate kinase M2 (PKM2) nuclear translocation [272]. It is noteworthy to indicate that, according to available literature, resveratrol could have opposite effects on angiogenesis, partially (but not exclusively) depending on the dose, stimulating the process at lower concentrations (<10 μM) and inhibiting it at higher ones (>20 µM) [273]. In a randomized placebo controlled clinical study, resveratrol did not affect prostate volume in healthy middle-aged men as measured by PSA levels and CT acquired prostate volumes, which does not support the use of this compound in the treatment of benign prostate hyperplasia [274].

A natural analog of resveratrol is piceatannol (Figure 8), a polyphenolic compound found in grapes, berries, peanuts and sugar cane. The generation of piceatannol occurs during fruit ripening and also during fermentation processes, being a common component in red wine [275]. In addition, during metabolism of resveratrol, piceatannol is produced by hydroxylation via cytochrome P450-1B1 enzyme [276]. Despite the chemical similarity between piceatannol and resveratrol (only differenced in an additional hydroxyl group) piceatannol exhibits a higher metabolic stability [277] pointing to the better ADME properties of this natural analog. Regarding the bioactive activities reported for piceatannol, this compound shows a preventive role in atherosclerosis, protecting against cardiovascular diseases. Furthermore, the antiaging, anti-inflammatory, antidiabetic, and antitumoral activities of piceatannol have been described [278]. Piceatannol exhibit a clear antiangiogenic potential in vitro and in vivo, and its mechanism of action is related to the inhibition of VEGF-A/VEGFR2 pathway [279], being a compound that should be taken into consideration for angiopreventive strategies.

Delphinidin (Figure 6) is a natural polyphenolic compound belonging to the anthocyanidine class. This blue-red pigmented compound is abundant in many fruits such dark grapes and berries, and vegetables, such as eggplants, red cabbages and tomatoes. Delphinidin and its natural occurring glycosides possess interesting health-promoting potential, exhibiting antioxidant, anti-inflammatory, antidiabetic and anticancer activity, among others [280]. The antiangiogenic activity of delphinidin has been reported in vitro and in vivo, and this effect seems to be mediated by a blockage of endothelial cell cycle (in G0-G1 transition point) and a decrease in VEGF-A/VEGFR2 signaling pathway [281,282]. In a tumoral context, delphinidin has been reported to decrease levels of EGF-induced VEGF-A expression in prostate and lung cancer cells, by a mechanism involving the inhibition of HRE-promoter activity in response to EGF induction, and the blockage of ERK and PI3K/Akt pathways [283,284].

Myricetin (Figure 6) is a flavonol found in vegetables (onions), fruits (grapes and berries), nuts and tea [285]. This compound shows multiple bioactive effects [286] that support its potential in clinical applications, such as anticancer therapy [210,287]. One of the anticancer mechanisms described for myricetin is related to its antiangiogenic effect [288]. Interestingly, myricetin possesses a backbone very similar to that of other flavonols that have been presented above, including quercetin, which seems to be related with a relevant antiangiogenic activity of the compound [289]. The role of myricetin as an angiogenesis inhibitor has been studied in endothelial cells, revealing a mechanism that involves induction of apoptosis and suppression of PI3K/Akt/mTOR signaling pathways [290], and a reduction in VEGF-A/VEGFR2 axis and in p38-MAPK survival pathway [291].

Quercetin is one of the most widely diffused flavonoid in fruits and vegetables in general, and is especially abundant in onion (*Allium cepa* L.), although is also rich in kale, broccoli, spinach, dill and oregano, among many other plant-derived foods [185,292,293]. It is an antioxidant and free radical scavenger and has anti-inflammatory and neuroprotective effects [294]. Quercetin inhibits proliferation, migration and tube formation in several endothelial cell lines [294,295,296,297,298], and blocks angiogenesis in the ex vivo rat aortic ring assay, and in the in vivo CAM assay, Matrigel plug assay and zebrafish model [295,298,299]. Interestingly, quercetin (20 mg/kg/d) is able to reduce tumor volume and weight in a prostate xenograft mouse model, affecting cell viability and apoptosis in prostate cancer cells. This has been correlated with the downregulation of Akt, mTOR and P70S6K pathways [296]. Likewise, quercetin suppresses VEGF-A induced phosphorylation of VEGFR2 and their downstream protein kinases in HUVECs [298,299]. In human retinal endothelial cells quercetin inhibits activation of VEGFR2 and Ras downstream cascade [294].

#### 4.2.4. Olives

The fruit of olive trees (*Olea europaea sativa*) is eaten as olives, but it is mainly consumed as olive oil, the most remarkable hallmark of the Mediterranean diet. Adherence to the Mediterranean diet has been associated to a lower risk of cancer mortality in the general population, as well as to a decreased risk of developing some types of cancer, including prostate and bladder cancer, what could be related to its antiangiogenic potential [300,301,302,303,304,305].

The olive leaves contain many phytochemicals, including oleuropein, ligustroside, oleacein, flavonoids and triterpenoids, and the majority of these compounds are still present in the olive fruit and olive oil, although in a lower proportion. Virgin olive oils, obtained exclusively by mechanical means, are those that best preserve the phytochemical content of the olives, which could be lost in the refinement process [306]. Olive phenolics are powerful antioxidants and could partially account for the observed health benefits of the Mediterranean diet [305,307]. The antiangiogenic properties of some of the bioactive compounds present in olive oil have been reported, showing a valuable angiopreventive potential [305].

The secoiridoid glycoside oleuropein (Figure 8) is an abundant phenolic compound in olive leaves, fruits and oil, being responsible for the bitter taste of olives. Due to the modulatory activity of oleuropein in multiple signaling pathways involved in cancer progression, the chemopreventive potential of this compound has been suggested [308,309]. The role of oleuropein as angiogenesis inhibitor has been evidenced in endothelial cells in vitro, leading to a reduction in cell growth and migration in response to VEGF-A induction, and interfering with the formation of tubular-like structures on Matrigel [310]. In an inflammatory context, oleuropein exhibits antiangiogenic activity through a mechanism involving the decrease in MMP-9 activity and the inhibition of PMA-induced COX-2 expression [311]. Of note, in olive ripening and during the processing of natural green olives, the endogenous hydrolysis of oleuropein by the enzymes β-glucosidase and esterase occurs, releases hydroxytyrosol [312], which has been suggested as a promising angiopreventive compound [305].

One of the most representative phenolic compounds in olives and extra virgin olive oil is hydroxytyrosol (Figure 8), derived from the hydrolysis of oleuropein by endogenous enzymatic activity during olive ripening. This phenolic alcohol behaves as a potent antioxidant and anti-inflammatory molecule. The therapeutic potential of hydroxytyrosol makes this natural compound one of the most promising chemopreventive agents present in the diet. Its antioxidant and anti-inflammatory effects support its multiple bioactivities in several prevalent pathologies, such as cancer, cardiovascular, neurodegenerative, and metabolic diseases [313,314,315,316,317]. Related to its antitumoral activity, hydroxytyrosol has been described as a potent angiogenesis inhibitor, both in vitro and in vivo, modulating the ECM remodeling potential of endothelial cells and inducing apoptosis [318,319]. In addition, the inhibition of VEGFR2 phosphorylation, and the downstream signaling events in ERK SAPK/JNK pathways seem to be implicated in the mechanism of action underlying the antiangiogenic effect of hydroxytyrosol [310]. Different semi-synthetic molecules derived from hydroxytyrosol have been described to retain and even improve the antiangiogenic activity of the original compound, supporting the promising use of hydroxytyrosol and derivatives in angioprevention [320,321]. Despite its antiangiogenic effect, the pro-angiogenic role of low doses of hydroxytyrosol has been recently reported in an in vitro model, suggesting a dose-dependent dual modulation of angiogenesis, which could be interesting for the treatment of ischemic injuries [322].

#### 4.2.5. Other Fruits

Among fruits, berries stand out for their particularly high levels of bioactive phytochemicals and vitamins [323]. Edible berries are being increasingly investigated for their potential in chemoprevention of a variety of chronic diseases, including cancer [324]. The antiangiogenic properties of some of the phytochemicals found in berries, such as resveratrol, piceatannol, some flavonoids and ellagitannins have been described above. Other interesting antiangiogenic compounds will be discussed now. Interestingly, whole berry extracts demonstrate more beneficial health effects in human studies than their individual components, probably due to their synergistic effects [323].

Fisetin (Figure 6) is a bioactive flavonol rich in strawberries. It can also be found in nuts, vegetables and fruits, such as tomatoes, onions and cucumbers, apples, mangoes, persimmons, kiwi, and grapes [325]. Among the multiple pharmacological properties described for fisetin, its anti-inflammatory and antioxidant activities are stand out, together with the inhibitory role of this compound in regulatory signaling pathways that support cancer progression [326,327]. The angiopreventive potential of fisetin results from its capability to target endothelial cells inhibiting angiogenesis in vitro and in vivo [328]. Actually, the antiangiogenic activity of fisetin has been evidenced in different tumoral contexts, such as retinoblastoma [329], melanoma [330], lung [331] and prostate cancer [332].

Berries, currants, grapes and some tropical fruits are rich in anthocyanins, colored water-soluble and glycosylated pigments belonging to the phenolic group, and responsible for the red, purple, and blue colors in fruits and vegetables. Red to purplish blue-colored leafy vegetables, grains, roots and tubers are the edible vegetables that contain a higher level of anthocyanin pigments. Among them, cyanidin-3-glucoside (Figure 6) is the major anthocyanin found in most of the plants [333]. of note, anthocyanin-rich extracts of several berries (wild blueberry, bilberry, cranberry, elderberry and strawberry) suppress hydrogen peroxide and TNF-α-induced VEGF-A expression in HaCaT cells (human keratinocytes), and delphinidin, cyanidin and malvidin (bilberry anthocyanidins) abrogate the VEGF-A-induced tube formation in endothelial cells co-cultured with fibroblasts [333,334,335]. Treatment with anthocyanin from wild blueberry decreases endothelial tube formation and downregulates Akt and eNOS gene expression [336]. Moreover, anthocyanin-rich extracts of *Hibiscus sabdariffa* inhibit angiogenesis in the CAM assay in a time- and concentration-dependent manner [337] and purple corn extract (rich in anthocyanins) antagonizes glomerular angiogenesis due to chronic hyperglycemia and diabetes by disturbing the angiopoietin-Tie2 ligand-receptor system linked to renal VEGFR2 signaling pathway [338].

Interestingly, cyanidin-3-glucoside impairs breast cancer angiogenesis both in vitro and in vivo by inhibiting STAT3/VEGF-A pathway. When HUVECs are treated with conditioned media from MDA-MB-231 and Hs-578T breast cancer cell lines, cyanidin-3-glucoside decreases tube formation in a dose-dependent manner and reduces MDA-MB-231 induced angiogenesis in the CAM assay [338]. The phenolic profile of purple açaí hydroethanolic extract reveals the presence of significant levels of anthocyanins, mainly cyanidin-3-glucoside, and other flavonoids. In vitro studies demonstrate that the mentioned extract exerts antiangiogenic activity with no cytotoxic effect and abrogates HMEC-1 migration and invasion, as well as their ability to form capillary-like structures [339].

**Figure 9:**
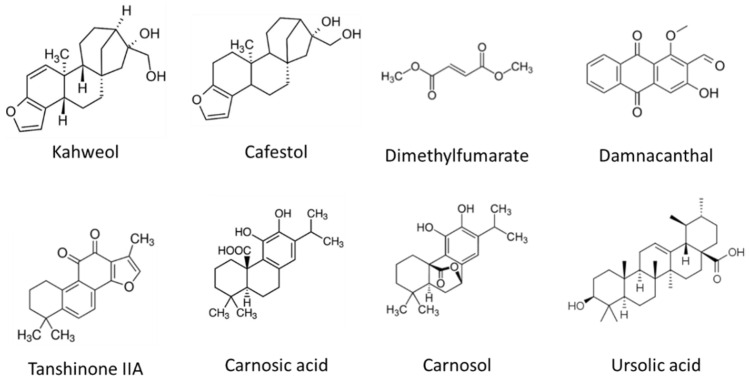
Chemical structures of some phytochemicals found in coffee (kahweol and cafestol), noni (damnacanthal), berries (ursolic acid), Danshen (tanshinone IIA), fumitori (dimethylfumarate) and rosemary (carnosic acid and carnosol).

Ursolic acid (Figure 9) is present in many berries, as well as in other edible fruits and plants, including apples, prunes, elder flower, peppermint, lavender, oregano and thyme. The role of this pentacyclic triterpene in cancer prevention and treatment is mediated by apoptosis induction and inhibition of cell proliferation, tumor angiogenesis and metastasis [340,341]. Ursolic acid inhibits different key steps of angiogenesis, including endothelial cell proliferation, migration, differentiation and proteolytic capability [342]. The inhibition of tumor angiogenesis by ursolic acid can be related to the suppression of multiple signaling pathways, leading to a decreased expression of VEGF-A, bFGF and iNOS genes. Furthermore, it suppresses the activation of sonic hedgehog (SHH), STAT3, Akt and p70S6K pathways, and decreases the proteolytic activity of endothelial cells by lowering the MMP-2 and 9 levels, while increasing those of TIMP-1 [343,344]. Despite the various interesting biological activities of ursolic acid, its pharmacological effect is limited by a low water solubility and difficulty in permeating some biological membranes [345]. This observation has fueled the development of new synthetic derivatives with enhanced therapeutic effects, bioavailability and absorption, which could improve their chemopreventive potential [346].

Noni, the fruit of *Morinda citifolia*, has been widely used in the traditional pharmacopoeias of native Hawaiians, other Pacific Islanders and Asian populations to treat various diseases [347]. After a successful marketing enterprise, it is now commonly taken by cancer patients as a dietary supplement, although, at the moment, there is little scientific evidence to support a significant effect on cancer prevention or treatment [348]. Very few low phase clinical studies have explored the activity of noni extract in cancer, including men with low-grade prostate cancer, but they did not result in any clear effect. More extensive clinical research are required to definitively clarify the real chemopreventive potential of this plant and that of the noni products that are already in the market [349,350].

Almost 200 bioactive phytochemical compounds have already been identified and isolated from different parts of the noni plant. Among them, the anthraquinone damnacanthal appears to be of significant biological importance. Damnacanthal (Figure 9) is a bioactive anthraquinone initially isolated from the phenolic phase of noni roots, although it is also present in its fruit, as well as in other *Rubiaceae* plants. Damnacanthal is an angiogenesis inhibitor with potential to reduce endothelial cell proliferation, impair sprouting in the ex vivo rat aortic ring explants, and abrogate blood vessel formation in the in vivo CAM assay, murine intradermal Matrigel plugs, and the transgenic *TGfli1:EGFPy1* zebrafish embryos [351]. Docking and molecular dynamics simulation approaches revealed that damnacanthal is a multikinase inhibitor affecting VEGFR2, c-Met and focal adhesion kinase. In vitro studies confirmed that this compound inhibits the phosphorylation of VEGFR1-3, and FGFR1, 2 and 4. Damnacanthal decreases protein levels and gene expression of MMP-2 and uPA of endothelial cells, what is in agreement with their defective migratory and invasive capabilities. The observed lowering of the adherence ability of damnacanthal-treated endothelial cell could be related to a decrease in their levels of integrin alpha 5 [351].

### 4.3. Beverages

#### 4.3.1. Tea

Tea is the most widely consumed plant-based beverage in the world. Very popular in Asian countries since ancient times as a daily drink and folk medicine, it was brought to England in the 17th century. Originated from the leaves of the evergreen plant *Camelia sinensis*, different types of tea can be obtained, depending on their production process, which changes the color of the leaves and their composition in phytochemicals. Among them, green tea is the one that contains more antioxidant molecules. Phytochemicals in tea have antimicrobial, diuretic, antidiabetic, antioxidant, chemopreventive, antiangiogenic and anti-inflammatory activities, making this beverage a potential aid in managing chronic diseases that are linked to lifestyle [352].

Some research suggests that green tea may also have a protective effect against various forms of cancer, including prostate cancer. Results from several population studies indicate that high consumption of green tea may prevent prostate cancer, contributing to the observed low prostate cancer mortality rates in Asian countries [353,354,355]. Several clinical trials have been devoted to elucidate the preventive role of tea and tea phytochemicals, which were well tolerated in clinical studies by disease-free men, men with precursor lesions and men with prostate cancer. In addition, the studies reported the detection of tea phytochemicals in prostate of treated patients, confirming their bioavailability. Although the relevance of these findings has been sometimes limited by the assay design, size or length, overall the randomized controlled trials have shown a decrease in serum PSA as well as a decreased progression rate in men with prostate cancer treated with tea phytochemicals [353,354,355]. Findings from two different studies with prostate patients that had been scheduled for radical prostatectomy, indicated that daily treatment with Polyphenon E, a tablet containing high concentrations of tea polyphenols, decreased the levels of several tumor markers, including circulating PSA, VEGF-A and IGF-1 [356,357]. In contrast, results from a phase II study with patients with androgen independent metastatic prostate carcinoma suggest that in patients with advanced prostate cancer, green tea may have limited benefits [358].

Although data regarding the preventive effect of tea and tea phytochemicals in other urologic cancers are scarce, findings from two meta-analyses revealed significant inverse associations between intake of green tea and black tea and the risk of bladder cancer [359,360]. Nevertheless, additional well designed studies are needed to fully confirm this chemopreventive effect [361].

Most of the polyphenols found in green tea belong to the catechins family, including epigallocatechin-3-gallate (EGCG), epigallocatechin, (−)-epicatechin-3-gallate and (−)-epicatechin. Among them, EGCG (Figure 6) is probably responsible for much of the cancer chemopreventive properties of green tea, is a powerful antioxidant, as well as an antiangiogenic, anti-inflammatory and antitumor agent capable to modulate tumor cell response to chemotherapy and induce apoptosis in cancer cells [362,363]. Antiangiogenic activity of EGCG, demonstrated in vitro and in vivo [364,365,366], can be mediated by inhibition of the VEGF-A expression, the binding of this growth factor to VEGFR2 or the phosphorylation of this receptor [367,368,369]. As for tumor cells, EGCG shifts the proteolytic balance of endothelial cells towards anti-proteolysis by downregulation of proteases and upregulation of their natural inhibitors. The antiangiogenic activity of this compound may be also related to a direct effect on the activation of HIF-1α and NFκB [370,371]. A small randomized, double blind, split face trial using a cream containing 2.5% *w*/*w* of EGCG demonstrated that the topical application of EGCG resulted in a decrease in both HIF-1α and VEGF-A expression in biopsies of volunteers with significant erythema and telangiectasia [372]. Other studies have shown that oral consumption of green tea by mice inhibits angiogenesis [366,373] in the corneal neovascularization model. Interestingly, some data suggest a selective effect of EGCG on tumor-associated endothelial cells and endothelial progenitor cells, responsible for tumor vasculogenesis, but not on normal endothelial cells [374]. EGCG suppresses liver metastases of human colorectal cancer and tumor growth in human pancreas xenograft mouse model [375].

#### 4.3.2. Coffee

Coffee is the third most consumed beverage in the world, after water and tea. The coffee plant was first cultivated in Africa, introduced by Muslims into Europe via Italy, and then into the Americas through French colonization. Coffee berries are dried once ripe, roasted at various temperatures to the desired flavor and then ground and brewed. The two most common species of coffee berries are *Coffea robusta* and *Coffea arabica* [376]. In addition to the presence of caffeine, as majoritarian bioactive compound in coffee, a high variety of molecules contained in this beverage has been reported to exhibit bioactive properties. They include other methylxanthines (theobromine, theophylline), diterpene alcohols (cafestol, kahweol), chlorogenic acids (affeoylquinic acids, feruloylquinic acids, p-coumaroylquinic acids), flavonoids (catechins, anthocyanins), hydroxycinnamic acids (ferulic acid, caffeic acid, p-coumaric acid), tocopherols and melanoidins [377]. Epidemiological studies suggest an inverse relationship between coffee consumption and prostate cancer risk, although this association remains controversial [233].

Although coffee was associated in the past with a higher risk of bladder cancer, recent studies revealed that smoking status was responsible for this carcinogenic effect [378]. Moreover, results from several studies have suggested that an increased coffee consumption could have a protective effect in bladder cancer [379,380,381].

Interestingly, some of the bioactive compounds present in the coffee have shown antiangiogenic properties, as is the case of the antioxidant diterpenes kahweol and cafestol (Figure 9) [382,383], supporting the potential role of coffee as angiopreventive beverage. The potent antiangiogenic activity of kahweol has been described in vitro, inhibiting key steps of the process, ex vivo (mouse aortic ring assay) and in vivo (CAM and zebrafish intersegmental vessel models) [382]. In addition, this compound also downregulates the expression of inflammatory molecules such as cyclooxigenase-2 (COX-2) and the monocyte-chemoattractant protein-1 (MCP-1) in endothelial cells, pointing to the interesting properties of kahweol as an anti-inflammatory molecule and to the possible implication of these targets in its antiangiogenic activity [382]. The antiangiogenic activity of cafestol has been also studied in vitro, revealing its ability to inhibit certain angiogenic steps, probably targeting Akt and FAK signaling pathways in endothelial cells [383].

The majority of diterpenes in coffee beans are esterified with different fatty acids, being the palmitate-esterified kahweol and cafestol the predominant forms [384,385,386]. The antiangiogenic potential of these two esterified diterpenes has been explored, revealing that kahweol and cafestol palmitate esters exhibit similar antiangiogenic properties than their free moieties in vitro. This can be due to a mechanism of action that involves downregulation of the Akt pathway through negative modulation of VEGFR2, presenting kahweol palmitate a more potent activity than the cafestol esterified form [387].

Although the antiangiogenic potential of these bioactive diterpenes supports the potential role of coffee as an angiopreventive beverage, an important issue about bioavailability has to be considered, since the content of kahweol, cafestol and other bioactive compounds in coffee is variable depending on the coffee species, the roasting process (temperatures and roasting time), and the brewing techniques (filtering of coffee is a critical step affecting the content of diterpenes). Attending to these factors, the traditionally unfiltered coffee consumed in Greece or Turkey retains a high amount of diterpenes, polyphenols and other bioactive compounds compared with filtered coffee [388], being its consumption associated with an improvement in endothelial function [389].

### 4.4. Herbs and Spices

Herbs and spices play important roles as flavoring agents, food preservatives and medicines for centuries. Herbal beverages, also called herbal teas, have long-since been used as therapeutic vehicles in traditional medicines, and currently are gaining increasing popularity among health-conscious consumers. Understood as aqueous infusions in hot or cold water of a given plant material, they extract an unspecified amount of the plant phytochemicals [390]. Although it is beyond the scope of this review to detail the myriad of phytochemicals that are present in herbs and spices, we will briefly mention some examples to illustrate their potential use in the angioprevention of urological tumors.

*Salvia miltiorrhiza Burge* (red sage or Danshen) has been used in traditional Asian medicine with preventive vascular properties [391]. The protective effects of Danshen on prostate cancer patients has been suggested by results from some retrospective studies in which a connection between the use of Danshen and survival was found [392]. Tanshinone IIA (Figure 9) is a natural terpenoid and the main bioactive component isolated from *Salvia miltiorrhiza* [393]. Tanshinone IIA exhibits antiangiogenic activity in HUVECs, by interfering the VEGF-A/VEGFR2 mediated activation of endothelial cells [394,395]. It inhibits the endothelial progenitor cells migration and tube formation by controlling the PLC, Akt and JNK signaling pathways [396]. The antiangiogenic activity of tanshinone IIA can also be mediated by a decrease in the proteolytic potential of endothelial cells, since this compound modulate the secretion of MMP-2 and TIMP-2 in an opposite way, resulting in the decreased MMP-2 activity of vascular endothelial cells [397]. The use of tanshinone IIA as an angiogenesis inhibitor is reinforced by virtue of its ability to inhibit the HIF-1α-mediated β-catenin/TCF3/LEF1 pathway in hypoxia and the TGF-β1-mediated β-catenin/TCF3/LEF1 pathway in normoxia [398].

Zyflamend is a hydroalcoholic extract of herbs including rosemary, turmeric, ginger, green tea, and oregano, among others. Normally used for antiinflammatory purposes, its anticancer potential has been suggested by a number of in vitro and in vivo studies [399,400,401,402]. Although some human studies have yielded promising results, supporting the chemopreventive potential of this herbal supplement in prostate cancer, more randomized clinical studies are needed to fully confirm its efficacy and clinical application [403,404,405].

Oregano is the name used to refer to up to 60 species of plants that share a particular flavor and odor [406]. *Origanum vulgare* L. is widely distributed in Europe and Asia, especially in the Mediterranean region. Dry oregano is used as a spice all over the work, and oregano teas have also been employed in folk medicine against cold, for digestive and respiratory problems [407]. The benefits for health of this plant have been attributed to a high content in phytochemicals, mainly flavonoid and phenolic acids [406,408]. The chemopreventive activity of oregano was studied in vitro and in vivo in a breast cancer model, showing antiangiogenic activity by a mechanism involving the reduction in VEGFR2 tumor expression [409]. Ethanolic extract of oregano mainly includes phenolic acids and flavonol derivatives, being rosmarinic acid (Figure 8) and luteolin (Figure 6) the two major components in the extracts [409]. The antiangiogenic effect of rosmarinic acid in vitro and in vivo has been reported [410,411], suggesting a mechanism of action targeting bFGF/FGFR axis [412]. Luteolin, a natural flavone, exhibits a potent antiangiogenic effect, suppressing VEGF-A stimulated angiogenesis steps in vitro and in vivo. The antisurvival effects of luteolin are mediated via blockage of PI3K/Akt-dependent pathways, whereas inhibition of the PI3K/p70 S6K pathway is responsible for the antimitotic effects of this compound [413]. Under chemically induced hypoxia, luteolin decreases the expression of VEGF and matrix metalloproteinase-9, suppresses the activation of HIF-1 and phosphorylated-signal transducer and activator of transcription 3 (STAT3) signaling [414]. This inhibitory activity has been assessed in different tumoral contexts in vivo (including prostate cancer) and in vitro, in which luteolin reduces tumor-associated vascularization [413,414,415,416].

Rosemary (*Rosmarinus officinalis*) is an aromatic evergreen shrub with narrow leaves that are used as spices and to make tea. It contains several bioactive compounds that are angiogenesis inhibitors, such as carnosol and carnosic acid (Figure 9). The antiangiogenic activity of these two diterpenes have been demonstrated in vitro and in vivo and it could be related to a mechanism inducing apoptosis in endothelial cells [417,418].

Fumitori *(Fumaria officinalis* L.) is an annual leafy plant used in the Kurdish and Europe ethnobotany for treatment of hepatobiliary dysfunction, gastrointestinal diseases, diuretic agents, cancer and skin disorders [419]. It contains some antiangiogenic phytochemicals previously described in this review, such as rosmarinic acid, quercetin and kaempferol, among others [420], but probably most of the angiopreventive potential of this plant derives from its content of fumaric acid esters. Dimethylfumarate exhibits interesting antiangiogenic properties, being able to interfere with certain functions of endothelial cells, including differentiation, proliferation, and migration. These results are reinforced by inhibition of in vivo angiogenesis, substantiated using CAM and live fluorescent zebrafish embryo neovascularization assays [421]. The antipsoriatic, antitumoral and antimetastatic activities of this compound might be mediated in part by antiangiogenic effects through the reduction in VEGFR2 expression [422].

The molecular targets of some of the antiangiogenic phytochemicals mentioned in this section are summarized in Table 2.

## 5. Conclusions. Limitations, Challenges and Future of the Use of Angiopreventive Strategies in Urologic Cancers

Lifestyle modification deserves consideration as a potential strategy of cancer prevention that could contribute to reduce health care costs and treatment-associated morbidities. Epidemiological data indicate that dietary habits may influence the risk of urologic cancer incidence and progression, suggesting a preventive potential of diet-based interventions involving fruits, vegetables and other plant-derived foods or dietary supplementals. Many of these plant-derived foods are rich in phytochemicals exhibiting a relevant antiangiogenic activity. Given the essential role that a deregulated angiogenesis plays at different stages of the tumor progression process, it seems foreseeable that many of these phytochemicals may be useful tools for cancer prevention and treatment.

Phytochemicals are derived from plants that have been used as food or in traditional medicine for centuries, what makes them particularly suitable for long-term use because of their reliability, low toxicity and affordability. Moreover, the fact that different phytochemicals often coexist in a given food product can increase the preventive efficacy without increasing toxicity and potentially reducing the emergence of resistance mechanisms. This is in agreement with the idea that the best results in antiangiogenic cancer therapy will come from the use of combination therapies, directed towards multiple targets in the tumor-endothelial microenvironment [109,134].

Nutritional recommendations to increase intake of fruits and vegetables can be easily assumed by a large part of the population, since they mirror those for cardiovascular and global health, showing, for example, that eating five servings a day of fruits and vegetables may help to achieve a healthier, longer life [423]. In this sense, the first level of angioprevention could build on preventive strategies already developed for other types of diseases (Figure 4).

Early detection could help to identify population at a higher risk for developing urologic cancer, which could benefit of a second level of angioprevention. A majority of urologic cancers are detected at early stages, either in a pre-neoplastic condition, or when they are slow growing and confined to the organ. This allows affected people to take an active role in making decisions as to what form of treatment they prefer to keep the cancer under control, while preserving quality of life. In addition, being involved in active surveillance can encourage these individuals to assume changes in their dietary habits or lifestyle in order to obtain a possible beneficial effect. This is in line with the results from the first large-scale phase III, randomized clinical trial of a behavioral intervention for a urological cancer demonstrating that a robust behavior modification promoting vegetable intake was feasible in patients with prostate cancer patients on active surveillance [424] (Figure 4). Finally, due to advances in diagnosis and treatment, more and more cancer patients have to cope with their illness for longer periods. For them, cancer becomes a chronic condition that must be controlled with treatments that are selected between patient and healthcare provider [425]. When making such a decision, both success probability and preservation of an acceptable quality of life are considered. The use of natural plant products and their derivatives can help to expand the armamentarium of less toxic antiangiogenic drugs aimed at improving the survival rates and the quality of life of cancer patients.

A limitation for the widespread application of angiopreventive strategies derives from the lack of robust clinical data to support their usefulness. Although the number of antiangiogenic drugs approved for cancer treatment is growing, cancer chemoprevention trials with dietary agents have often achieved limited clinical success. Regardless of the number of studies, nutrition science frequently shows inaccurate and/or contradictory results, due to a series of limitations that make difficult the study of long-term illnesses by means of clinical trials. In many cases, the results were derived from observational studies based on inaccurate and unrealistic dietary surveys, where uncontrolled factors could have distorted the outcome of the investigations. Well-designed clinical trials are needed to establish optimal conditions for cost-effective and readily applicable chemoprevention using dietary phytochemicals. These will require a proper stratification of the individuals involved in the study, targeting high-risk individuals instead of larger populations at relatively low risk to develop cancer. This would allow to achieve favorable benefit-risk ratios, providing more definitive results with less effort [18]. In the case of angiopreventive strategies, the finding of biological markers to determine the degree of response to a therapeutic strategy, as well as to identify which subjects will most likely benefit from a given intervention, still remains a major clinical challenge [426].

Attention should also be paid to some factors such as gut microbiota composition or gene-nutrient interactions, which could have an impact on the response to a given food by different subjects [427,428,429], or the nutritional profiles of the food, which could be affected by their handling and storage [428,430,431,432]. In this regard, minimizing the cooking or processing of plant-derived foods, could be a good general advice to preserve their phytochemical contents, whereas wealthiest regions could also afford preventive interventions based on the use of plant extracts, nutritional supplements or isolated phytochemicals. This could help to better select the dose, formulation and schedule of the intervention material [433]. In addition, this kind of strategies could awaken the interest of pharmaceutical companies in the development of new molecules that, based on their natural phytochemical counterpart, could improve their pharmacokinetic or pharmacodynamic properties [147].

If we have learned anything from the COVID-19 global pandemic, it is that results can be achieved in a short period of time, as long as forces are united and sufficient resources are provided. As far as cancer chemoprevention in general and angioprevention in particular are concerned, success in the short to medium term will depend on combining the efforts of basic and clinical research, supported by increased funding from public agencies, and from the food and pharmaceutical industries. For sure, these efforts will be rewarded in the future by an improvement in the quality of life and survival of cancer patients, achieved at an affordable cost.

## Figures and Tables

**Table 1:** Antiangiogenic drugs approved by the FDA for the treatment of solid tumors.

Drug	Type	Malignancies	Molecular Target	Year of First Approval
Axitinib (Inlyta^®^)	TKI	Advanced RCC	VEGFR1-3, PDGFRβ	2012
Bevacizumab (Avastin^®^)	Humanized monoclonal antibody	MCRC, NSCLC, OC, MBC, glioblastoma, metastatic RCC, endometrial cancer, mesothelioma and cervical cancers	VEGF	2004
Cabozantinib (Cometriq^®^ and Cabometyx^®^)	TKI	Refractory advanced RCC, metastatic medullary TC and PNET	VEGFR2, Tie2	2012
Everolimus (Afinitor^®^)	TKI	RCC, GIST, lung carcinoma, advanced breast cancer, PNETs and sub-ependymal giant cell astrocytoma	mTOR	2009
Lenalidomide (Revlimid^®^)	Amino acid	Myeloma (MDS) and mantle cell lymphoma	VEGF, bFGF	2005
Lenvatinib mesylate (Lenvima^®^)	TKI	TC, HCC and RCC	VEGFR1-3, PDGFRα, FGFR1-4	2015
Nintedanib (Vargatef^®^ and Ofev^®^)	TKI	Idiopathic pulmonary fibrosis	VEGFR1-3, PDGFR, FGFR1-2	2014
Pazopanib (Votrient^®^)	TKI	Metastatic STC and advanced RCC	VEGFR1-3, PDGFRβ, FGFR1-2	2009
Pomalidomide (Pomalyst^®^ and Imnovid^®^)	Amino acid	Multiple myeloma	VEGF, IL-6, COX-2, Cereblon	2013
Ramucirumab (Cyramza^®^)	Humanized monoclonal antibody	MCRC, NSCLC, and advanced gastric adenocarcinoma	VEGFR2	2014
Regorafenib (Stivarga^®^)	TKI	Chemo-refractory MCRC, unresectable HCC and GIST	VEGFR1-3, PDGFRβ, FGFR1-2	2012
Sorafenib (Nexavar^®^)	TKI	Advanced RCC, metastatic differentiated TC and unresectable HCC	VEGFR2, PDGFRβ	2005
Sunitinib (Sutent^®^)	TKI	Metastatic RCC, GIST, PNET and TC	VEGFR1-2, PDGFRα/β	2006
Thalidomide (Thalomid^®^)	Amino acid	Multiple myeloma in combination with dexamethasone	VEGF-A bFGF, FGFR	2006
Temsirolimus (Torisel^®^)	TKI	RCC	mTOR	2007
Vandetanib (Caprelsa^®^)	TKI	Unresectable or metastatic TC	VEGFR2	2011
Ziv-Aflibercept (Zaltrap^®^)	Fusion protein (VEGFR chimera)	MCRC in combination with 5-FU, irinotecan and leucovorin	VEGF-A/B, PlGF	2012

TKI (tyrosine kinase inhibitor), MCRC (Metastatic colorectal carcinoma), NSCLC (non-small-cell lung cancer), OC (ovarian cancer), MBC (metastatic breast cancer), RCC (renal cell carcinoma), HCC (hepatocellular carcinoma), TC (thyroid carcinoma), STC (soft tissue carcinoma), GIST (Gastrointestinal Stromal Tumors), MSD (myelodysplastic syndrome), PNET (pancreatic neuro-endocrine tumors). Source: Drugs@FDA: FDA-Approved Drugs, https://www.accessdata.fda.gov/scripts/cder/daf/ accessed on 22 November 2021.

**Table 2:** Antiangiogenic effect of some selected phytochemicals, mentioned in this review.

Name	Structure	Source	Possible Mechanism	References
Damnacanthal	Anthraquinone	Noni	↓VEGFR1-3, c-Met, FAK, FGFR1, 2 and 4 ↓MMP-2, uPA and integrin α5 ↓PI3K-Akt signaling pathway	[351]
Delphinidin	Polyphenol	Grapes, berries, eggplants, red cabbages and tomatoes	↓VEGF-A/VEGFR2 pathway ↓VEGF-A expression ↓PI3K-Akt and ERK signaling pathways	[280,281,282,283,284]
Epigallocatechin gallate	Polyphenol	Teas, berries, kiwis, cherries, pears, peaches, apples, nuts and herbs	↓VEGF-A and MMP-2 expression ↓ VEGF-A/VEGFR2 pathway ↓HIF-1α, NFκB	[363,364,365,366,367,368,369,370,371,372,373,374,375]
Genistein	Polyphenol	Soybeans	↓VEGF-A, PDGF, MMPs expression ↑angiogenesis inhibitors ↓HIF-1α, PI3K/Akt, MAPK, NFκB and Bax/Bcl-2	[194,203,204,205,206,207,208,209]
Hydroxytyrosol	Phenolic alcohol	Tomato, pink grapefruit, oranges and watermelon	↓VEGFR2 phosphorylation and ERK SAPK/JNK pathways ↓MMPs secretion ↓PI3K/Akt and NFκB ↑Caspases 3 and 7 activation	[305,313,314,315,316,317,318,319,320,321,322]
Kaempferol	Polyphenol	Cruciferous vegetables, spinach, onions, leeks, citrus fruits, grapes and herbs	↓VEGF-A expression and secretion ↓HIF-1α, VEGF-A/VEGFR2 pathways ↓PI3K/Akt, mTOR and MEK/ERK	[186,187,188,189,190]
Luteolin	Polyphenol	Oregano	↓VEGF-A/VEGFR2, p38/MAPK and PI3K/Akt/mTOR signaling pathways ↓VEGF-A and MMP-9 expression ↓HIF-1α and STAT3	[413,414,415,416]
Lycopene	Carotenoid	Tomatoes, pink grapefruit, oranges and watermelon	↓MMP-2 and uPA ↓VEGFR2-mediated PI3K/Akt and ERK signaling pathways	[216,217,218,219,220,221]
Myricetin	Polyphenol	Onions, grapes, berries, nuts and herbs	↓VEGF-A/VEGFR2, p38/MAPK and PI3K/Akt/mTOR signaling pathways	[286,287,288,289,290,291]
Piceatannol	Polyphenol	Grapes, berries, peanuts and sugar cane	↓VEGF-A/VEGFR2- mediated pathways	[279]
Quercetin	Polyphenol	Onions, cruciferous vegetables, spinach, apples, berries, nuts, oregano, teas and herbs	↓VEGF-A/VEGFR2, p38/MAPK, Akt, mTOR and P70S6K signaling pathways	[292,293,294,295,296,297,298,299]
Resveratrol	Polyphenol	Grapes and other fruits (mainly berries) and nuts	↓VEGF-A expression ↓HIF-1α, GSK3b/β-catenin/TCF, VEGF-A/VEGFR2 and TFG-β pathways ↓ERK-mediated PKM2 nuclear translocation	[266,267,268,269,270,271,272,273]
Rosmarinic acid	Phenolic acid	Oregano, lemon balm, sage, marjoram and rosemary	↓bFGF/FGFR signaling pathway	[410,411,412]
Sulforaphane	Glucosinolate	Cruciferous vegetables (mainly broccoli)	↓HIF-1α, VEGF-A/VEGFR2 pathways ↓MEK/ERK and PI3K/Akt	[182,183,184]
Tanshinone IIA	Terpenoid	Danshen	↓VEGF-A/VEGFR2 and PI3K/Akt pathways ↓MMP-2 and ↑TIMP-1 levels ↓HIF-1α-mediated β-catenin/TCF3/LEF1 pathway in hypoxia ↓TGF-β1-mediated β-catenin/TCF3/LEF1 pathway in normoxia	[393,394,395,396,397,398]
Ursolic acid	Pentacyclic triterpene	Berries, apples, prunes, elder flower, peppermint, oregano and herbs	↓MMP-2 and 9, ↑TIMP-1 levels ↓VEGF-A, bFGF and iNOS genes expression ↓Activation of SHH, STAT3, Akt and p70S6K pathways	[340,341,342,343,344,345]

## Data Availability

Not applicable.
